# Cultural transmission, networks, and clusters among Austronesian-speaking peoples

**DOI:** 10.1017/ehs.2024.45

**Published:** 2024-12-06

**Authors:** Joshua C. Macdonald, Javier Blanco-Portillo, Marcus W. Feldman, Yoav Ram

**Affiliations:** 1 School of Zoology, Faculty of Life Sciences, Tel Aviv University, Tel Aviv, Israel; 2 Department of Biology, Stanford University, Stanford, CA, USA; 3 Safra Center for Bioinformatics, Tel Aviv University, Tel Aviv, Israel

**Keywords:** cultural evolution, Austronesian, Polynesian, Oceania, cultural networks

## Abstract

With its linguistic and cultural diversity, Austronesia is important in the study of evolutionary forces that generate and maintain cultural variation. By analysing publicly available datasets, we have identified four classes of cultural features in Austronesia and distinct clusters within each class. We hypothesized that there are differing modes of transmission and patterns of variation in these cultural classes and that geography alone would be insufficient to explain some of these patterns of variation. We detected relative differences in the verticality of transmission and distinct patterns of cultural variation in each cultural class. There is support for pulses and pauses in the Austronesian expansion, a west-to-east increase in isolation with explicable exceptions, and correspondence between linguistic and cultural outliers. Our results demonstrate how cultural transmission and patterns of variation can be analysed using methods inspired by population genetics.

## Graphical abstract

**Figure:**
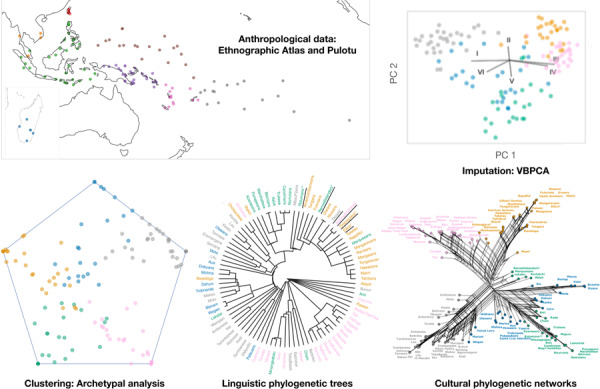

**Social media summary:** We identify clusters of cultural traits and differences in modes of transmission among traits in Austronesian cultures.

## Introduction

The origin and evolution of patterns of variation in human cultures and non-human animal traditions have been subject to intensive study over the past six decades (Marler & Tamura, 1964; Slater & Ince, 1979; Whiten et al., 2007; Creanza et al., 2015; Garland & McGregor, 2020; Zandberg et al., 2021; Turchin et al., 2022; Skirgård et al., 2023; Whitehouse, 2023). This research has often been framed in terms of expected differences between patterns of genetic and cultural variation (Ammerman & Cavalli-Sforza, 1973; Cavalli-Sforza & Feldman, 1973, 1981; Feldman & Cavalli-Sforza, 1976; Rindos et al., 1985; Creanza et al., 2015). The distribution of cultural variation among human populations is not expected to be random, and an important historical and anthropological question is what forces shaped the patterns of cultural diversity within and between populations. While genetic inheritance is predominantly vertical, at the macro scale, cultural variants may be transmitted vertically or copied/adopted non-vertically via mechanisms such as learning, exchange, conquest and competition (Dyson-Hudson & Smith, 1978; Marcus, 2008; Karin & Alon, 2018; Turchin & Gavrilets, 2021). Both geography and genealogy are likely to contribute to the variation observed in some cultural datasets. For example, decades ago Ammerman and Cavalli-Sforza (1973) investigated whether the spread of farming from the Middle East to Europe was due to vertical processes such as the migration of farmers themselves, as reflected in genetic patterns, or the non-vertical transmission of the farming culture without the necessity of human migration. Similarly, Jeong et al. (2018) found that the spread of dairy pastoralism in the eastern steppes was likely to have occurred through adoption by local hunter–gatherers rather than population replacement. Patterns of cultural variation in practices, beliefs or social structures may be analysed statistically to estimate how cultural traits covary in terms of geographical proximity or other relationships among populations. For example, Turchin et al. (2018) used logistic regression, principal components analysis (PCA) and metrics of different aspects of cultural complexity and showed a generally increasing trend in sociopolitical complexity through time and across geographic regions.

Cultural microevolution can be defined as a change in the frequency of cultural traits within a single population (Turchin & Gavrilets, 2021). In this framework, vertical transmission is defined as individuals acquiring a trait from their parents. Processes of non-vertical transmission occur when individuals acquire traits from non-parental adults or peers of the same generation (Cavalli-Sforza & Feldman, 1981; Creanza et al., 2017). In contrast, cultural macroevolution is focused on change in cultural traits in meta-populations (Eldredge, 2009; Turchin & Gavrilets, 2021). From a macro-evolutionary perspective, the ‘tempo’ of evolution refers to evolutionary rates and their variation in time and space. The ‘mode’ of evolution relates to mechanisms of both vertical (e.g. migration) and non-vertical (e.g. competition, exchange, conquest) cultural change at the societal scale (Dyson-Hudson & Smith, 1978; Marcus, 2008; Karin & Alon, 2018; Turchin & Gavrilets, 2021). At the population scale, vertical transmission can be defined as the flow of traits from ‘parent’ to ‘daughter’ cultures (Turchin & Gavrilets, 2021).

Oceania is an informative region for studying human ecology and cultural evolution owing to its cultural and linguistic diversity, relatively recent contact with outside cultures (e.g. Europeans) and the availability of a large body of relevant ethnographic literature (Kirch, 2017; Sheehan et al., 2023). Austronesian-speaking people spread throughout Oceania after leaving Taiwan around 5000–3500 YBP (years before present), ultimately reaching the islands of Hawai'i (900 YBP), Aotearoa (New Zealand) and Rapa Nui (Easter Island) from 600 to 800 YBP (Chambers & Edinur, 2021; Ioannidis et al., 2021), and as far west as Madagascar (1500 YBP, Bellwood et al., 2011; Chang et al., 2015). It has been suggested that Austronesian maritime technologies allowed the exploration of new frontiers and settlement of small and large islands distant from neighbours and the mainland (Spriggs, 1995; Bellwood et al., 2011; Kirch, 2017).

Many aspects of cultural variation have been analysed for Austronesian societies. Rogers and Ehrlich (2008) and Rogers et al. (2009) investigated how structural and decorative variation in canoes used by Oceanic peoples (Haddon & Hornell, 1975) may have been subject to selection as opposed to stochastic cultural phenomena analogous to genetic drift. The observed patterns suggested that canoe decorations may be more subject to random (undirected) change than structural features, which may have been subject to selection. However, the latter is difficult to separate from the influence of biases in cultural transmission. Indeed, neither structural nor decorative canoe variation fit a genealogical pattern (Gray et al., 2010). Karin and Alon (2018) took a different approach, analysing Austronesian cultural variation using Pareto task inference (Shoval et al., 2012). They found three ‘ecotypes’, which they called *resource competition*, *mobility/exchange* and *resource defence*, aligned with three of the four clusters suggested by Dyson-Hudson and Smith (1978) in the context of economic-dependability theory. Gray et al. (2009) constructed a linguistic phylogeny of over 400 Austronesian groups, implicitly assuming some degree of vertical transmission. Indeed, Gray et al. (2010) found that the phylogeny of Austronesian languages in Papuasia and Polynesia had a ‘moderately tree-like’ structure, which can be interpreted as the result of cultural vertical transmission. Subsequent studies have used this phylogeny, together with other evolutionary frameworks, to investigate the tempo of co-evolution in cultural traits such as ritual human sacrifice (Watts et al., 2016), social stratification and agricultural intensification (Watts et al., 2016; Sheehan et al., 2018), systems of political and religious authority (Sheehan et al., 2023), and belief in supernatural punishment and moralising high gods (Watts et al., 2015a) assuming mostly genetic-like vertical transmission across the phylogeny.

Different kinds of cultural traits may evolve in different ways. Guglielmino et al. (1995) considered models incorporating demic diffusion, environmental adaptation and cultural diffusion, and tested the fit of these models to the patterns of variation for different cultural traits – finding that each model best explained the variation in some cultural traits, and that traits related to kinship patterns tended to be conserved across generations. Similarly Currie and Mace (2014) found that while different cultural traits tend to evolve at similar rates in different geographic regions, there are differences in evolutionary rates between cultural traits related to external environmental conditions and those related to social structure.

**Figure 1.**
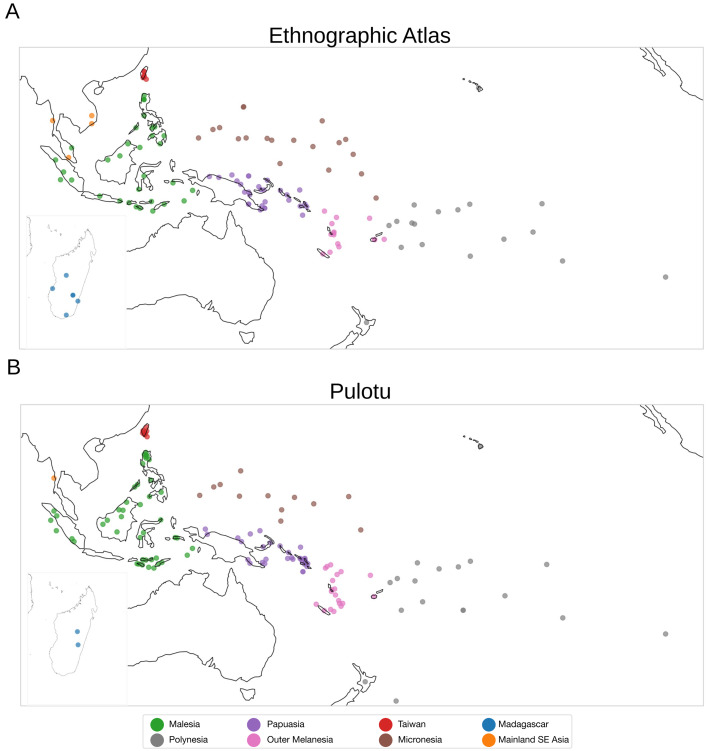
Austronesian cultures in this study. Geographic locations of cultures in (a) the Ethnographic Atlas and (b) Pulotu. See the supplementary data for a complete list. Malesia combines Indonesia and the Philippines; Papuasia combines New Guinea, its surrounding islands and the Solomon Islands; Outer Melanesia combines Vanuatu and New Caledonia.

Here, we study two cross-cultural datasets, *Pulotu* and the *Ethnographic Atlas* (Murdock, 1967; Watts et al., 2015b; Kirby et al., 2016). Teams of subject-matter experts encoded the cultural variables in these datasets based on previous ethnographies. Pulotu includes 137 Austronesian cultures and was compiled for studying Religion in Austronesian people (Watts et al., 2015b; Kirby et al., 2016). Pulotu also contains data on other aspects of culture and several geographical and historical features, such as island type and the presence or absence of pre-Austronesian populations. We focus on 36 variables from Pulotu that are informative on traditional/indigenous cultural traits and conflict within and between groups and are missing no more than 50% of their entries (Supplementary Data). The Ethnographic Atlas (EA) includes 1291 cultures from around the world (Murdock, 1967; Kirby et al., 2016) and many cultural variables divided into subsistence, labour, community organisation and kinship practices, among others. We focus on 69 EA variables (Supplementary Data) and the 130 groups of people that speak Austronesian languages (Figure 1).

The variables in Pulotu and EA are either categorical or ordinal: an example from Pulotu is *degree of external conflict*, which takes values between 1 (*frequent external conflict*) and 4 (*absence of external conflict*). Multiple categories were assigned to each feature in the original data. Based upon these category assignments, we identify four classes of cultural variables from the resulting broad classifications (Table 1): (i) *social organisation*; (ii) *subsistence* (both from EA); (iii) *religion*; and (iv) *cultural interaction* (both from Pulotu). Previous studies of Austronesian cultural evolution have focused on covariation of two or three cultural features (cf. Watts et al., 2016; Sheehan et al., 2018, 2023), aggregated all cultural features (cf. Karin & Alon, 2018) or focused on linguistic variation as a proxy for total cultural variation (cf. Gray et al., 2009, 2010; Padilla-Iglesias et al., 2020). Seeking a unified approach, we divided the data into cultural classes with three questions in mind: (i) did the modes of transmission differ among these cultural classes; (ii) were there distinct patterns of variation for each of these cultural classes; and (iii) is geography alone sufficient to explain (some) of these patterns of variation?

In analysing Pulotu and EA, we first deal with missing data. Cultural data may be incomplete owing, for example, to non-standardised data collection methods in ethnography and archaeology (Bernard & Gravlee, 2014; Hester et al., 2016) and the variable degree of preservation of material culture in the archaeological record. Therefore, we imputed missing values in the data. Furthermore, cultural traits may be transmitted in linked packages, which should be accounted for in the inference of cultural transmission (Yeh et al., 2019). We analysed patterns of cultural covariation using *archetypal analysis*. This is a computational method for dimension reduction and soft-clustering. It represents each data point as a convex combination of representative points, i.e. ‘archetypes’ (Cutler & Breiman, 1994; Mørup & Hansen, 2012). Archetypal analysis and conceptually similar methods, such as *ADMIXTURE* and *STRUCTURE*, have been widely applied in population genetics and genomics (Pritchard et al., 2000; Li et al., 2008; Alexander et al., 2009; Alexander & Lange, 2011; Gimbernat-Mayol et al., 2022). After identifying cultural clusters, we assessed potential differences in their cultural transmission. We calculated the pairwise euclidean distance between cultures in the archetype space, which accounts for covariance in cultural traits in a way that distances calculated in the trait space do not, and for potential sources of over- or under-estimating similarity. Next, we constructed cultural phylogenetic networks (Bryant & Moulton, 2004; Huson et al., 2008) from the pairwise distances and then assessed the ‘tree-likeness’ of these phylogenies – or the degree to which they were generated by a genealogical process with vertical transmission (Holland et al., 2002; Huson et al., 2008; Gray et al., 2010).

In the cultural macroevolution of Austronesia, we find correspondence between cultural and linguistic outliers and differing degrees of vertical transmission among the cultural classes, with *social organisation* and *subsistence* being relatively more vertically transmitted than *religion*. We also find a clear geographic explanation for variation in the *cultural interaction* cultural class. These results suggest that *social organisation* and *subsistence* are more determined by cultural continuity between subsequently settled islands than *religion*. However, none of the cultural classes show variation determined by vertical transmission. In the *social organisation* class, we find that lineal biases in residence patterns, kinship reckoning and inheritance rules are not always parallel. In the *subsistence* class, we find support for previously suggested pauses in migration and shifts in the mode of subsistence that appear to correspond with these pauses. We also find that grain agriculture is not a prerequisite for the rise of complex societies. Our results demonstrate that analyses inspired by population genetics can quantify and illuminate the complex processes of cultural transmission, ecology and geography that jointly generate observed patterns of cultural variation.

## Methods

### Imputation and dimension reduction

To address the problem of missing data, we first perform *variational bayesian principal components analysis* (VBPCA; Ilin & Raiko, 2010b) previously implemented in Matlab (Ilin & Raiko, 2010a). This probabilistic version of PCA, in contrast to traditional imputation approaches, such as multivariate iterative imputation (Van Buuren and Oudshoorn, 1999), reconstructs the entire dataset, estimates posterior distributions for both the data and principal components, filters noise in the data and performs dimension reduction (Figures S18–S21). We imputed features with values present for more than 50% of the cultures (rows) and removed features with values present for less than 50%.

We compared the performance of VBPCA to iterative imputation with regression implemented in *Scikit-learn* (Pedregosa et al., 2011). For the initialisation step, where a first estimate is obtained, missing values were replaced by the column median. We considered the following regression models: bayesian ridge regression, nearest-neighbours regression with five or 10 neighbours, decision tree regression and random forest regression with 100 trees. We found that the best iterative imputation model and VBPCA produced similar results (Figures S18 and S20). Therefore, we elected to use VBPCA because it provides explicit marginal posterior distributions for the data and the principal components and performs dimension reduction, similarly to standard PCA.

### Cluster analysis

#### Archetypal analysis

Archetypal analysis represents samples in a dataset as a convex combination of extreme points or ‘corners’ (Cutler & Breiman, 1994; Mørup & Hansen, 2012; Gimbernat-Mayol et al., 2022). Given an *n* × *p* dimensional data matrix *X* composed of *n* samples and *p* features, and *k* archetypes, archetypal analysis approximates1
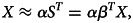
where *α* is a non-negative *n* × *k* matrix of archetype weights in which each row sums to 1, *β* is a sparse non-negative *n* × *k* matrix in which each column sums to 1, and *S* = *X*^*T*^*β*. is a *p* × *k* loadings matrix. Then, an optimisation algorithm is used to find the *α* and *β* that minimise the approximation error ||*X* − *αβ*^*T*^*X*|| (Mørup & Hansen, 2012); we use the archetypal analysis algorithm previously implemented in the Python package pypcha (Aslak, 2016). Notably, *α*_*i*,*j*_ can be interpreted as the contribution of archetype *j* to population *i*, or, for soft-clustering analysis, as the membership coefficient of population *i* in cluster *j*.

In our analysis, *X* is the mean-centred, imputed, and noise-filtered data matrix reconstructed using VBPCA. The following section explains how we determine the number of archetypes, *k*.

#### Number of archetypes

Patterson et al. (2006) have previously developed a method to determine the number of ‘large’ eigenvalues in a PCA decomposition, which they expect to equal *k* − 1. Their method makes several assumptions about the dataset (e.g. the number of rows is much lower than the number of columns, and data entries are identically and independently distributed) that may be unsuitable for cultural datasets. Recently, we have extended their method to apply to datasets with missing data without constraints on the number of rows vs. columns or assuming an identical distribution of data entries. Briefly, we apply the above-mentioned VBPCA approach and use the posterior distribution of the reconstructed data matrix to develop a significance test, similar to the TW test of Patterson et al. (2006), which does not assume a theoretical distribution for the eigenvalues (i.e. the TW distribution) but instead uses a resampling approach. In Macdonald et al. (2024b), we provide full details of the approach, validate it using randomly generated datasets and demonstrate its application to several datasets.

#### Clustering in the archetype space

It is possible to have more clusters than archetypes in population genetics and genomics, for example, if individuals have similar mixtures of two or more archetypes (Behr et al., 2016). Thus, we performed K-means clustering for each cultural class to determine if such mixtures exist.

We considered three different metrics to score the results of K-means clustering: the *silhouette score* (Rousseeuw, 1987) measures how similar an average data point is to its cluster (higher is better); the *variance ratio criterion* (Caliński & Harabasz, 1974) is the ratio of the sum of between-cluster dispersion to within-cluster dispersion (higher is better); the *Davies–Bouldin index* (Davies & Bouldin, 1979) is defined as the average similarity of each cluster to its most similar cluster – further apart and less dispersed clusters will result in a lower index value (lower is better).

For each cultural class, we considered any number of clusters between two less and two more than the number of archetypes, except for *cultural interaction*, where we considered between two and four clusters (as the number of archetypes is two). We set the number of clusters to be a value for which two or more of the above metrics were optimised; if no such value exists, we set it to the number of archetypes, *k* (Figure S12).

### Outlier detection

We first test whether there are significant differences in regional archetypes (see Figure 1 for the geographic regions). We examine the archetype matrices for each pair of regions, in which each row records the archetype weights of a single culture in that region. We use maximum likelihood to fit two models, *M*_0_ and *M*_1_, to these archetype matrices. The null model, *M*_0_, assumes both matrices are independent samples from the same Dirichlet distribution, a natural distribution for modelling archetype weights. Fitting this model requires estimating the parameters of a single distribution. The alternative model, *M*_1_, assumes each matrix is a sample from a distinct Dirichlet distribution. Fitting this model requires estimating the parameters of two distributions, one for each matrix. We then perform a chi-square test on the likelihood ratio of the two models to determine if the null model can be rejected in favor of the alternative model, from which we can conclude that the two regions are significantly distinct. This process was previously implemented in the test function of the Python dirichlet package (Minka, 2000).

Next, using the following steps, we test if Polynesia provides a better explanation for the outlier archetype weights than the actual geographic region for each cultural class and each known Polynesian outlier (see above) in a region significantly distinct from Polynesia. We first sample *N* datasets from the posterior distribution of the VBPCA data reconstruction and apply archetypal analysis to each sample. Then, for each geographic region, we fit a Dirichlet distribution to the region archetype matrix using maximum likelihood, as above (this is performed *N* times per region). Next, for each Polynesian outlier, we computed the log-likelihood of its archetype weights under the fitted Dirichlet distribution for both Polynesia and the actual region. For each Polynesian outlier, we have *N* log-likelihoods for Polynesia and *N* log-likelihoods for the actual region. We then perform the Mann–Whitney *U*-test on these two sets of log-likelihoods under the null hypothesis that the distributions of log-likelihoods for the two regions are the same, with the alternative hypothesis that the distribution of log-likelihoods for Polynesia is stochastically dominant over the distribution of log-likelihoods for the actual region. If the null hypothesis is rejected (*p*-value < 0.05), then we conclude that the Polynesian outlier is also a cultural outlier (Tables S8 and S9).

Finally, to examine differences in the intra-regional variance between cultures, we compared variation within regions in the archetype space using a normalised *F*_*st*_,. metric, which is strongly correlated with the Dirichlet distribution variance, using the R package fststruct (Morrison et al., 2022; Tables S2–S6, Figure S10).

### Cultural distance, phylogenetic networks and mechanisms of cultural transmission

To assess the extent to which the observed archetype variation agrees with a process of vertical cultural transmission, a pairwise distance matrix for each cultural class is constructed by calculating the distance *d*(*i*, *j*) between cultures *i* and *j* as the Euclidean distance in the archetype space (Figure S13).

**Figure 2.**
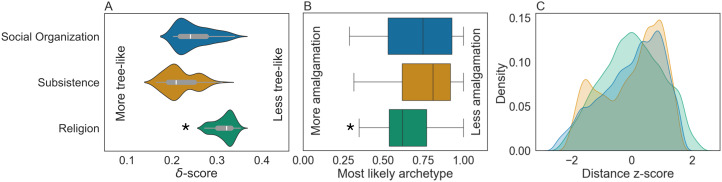
Relative tree-likeness of cultural classes. (a) *Religion* has higher *δ*-scores and is, therefore, less tree-like than *social organisation* and *subsistence* (mean *δ*-scores: 0.32, 0.25 and 0.22, respectively). (b) Boxplots of the membership coefficient (,-*i*;*j*.) of the most likely archetype of each culture illustrate the degree of cultural amalgamation in each cultural class. Asterisks denote statistically significant differences (Table S10). (c) Kernel density estimate plots for the normalised pairwise distances. *Religion* has a symmetrical distribution of cultural archetype distances between cultures, whereas the other cultural classes have skewed/multi-modal distributions (Fisher–Pearson skew coefficient −0.07, −0.48 and −0.43, respectively; Hartigan dip-test of unimodality statistic (Hartigan & Hartigan, 1985) 0.004, 0.011 and 0.011, with *p*-values = 0.12, 0, and 0, respectively).

We then use the Neighbor-Net algorithm (Bryant & Moulton, 2004) to construct a phylogenetic network from this pairwise distance matrix using the splitstree software package (Huson et al., 2008). The phylogenetic networks are then visualised using splitstree (Figures 2, S4 and S5). These networks may be more or less tree-like, and the number of reticulations provides a visual representation of the degree to which a network is generated by non-vertical transmission processes, with more reticulations indicating less verticality.

We also computed the *δ*-scores and *Q*-residuals for each distance matrix (Figure 2) to quantify the tree-likeness of each cultural class (Holland et al., 2002; Huson et al., 2008). These metrics measure the extent to which taxa in the phylogenetic network satisfy the *four-point condition* (Steel, 2016), with a low score indicating a tree-like network mostly owing to vertical transmission and a high score indicating non-tree-like networks, probably owing to the action of parallel evolution or non-vertical (e.g. horisontal) transmission (Gray et al., 2010). In the extreme case, as with the binary *Cultural Interaction* archetypes, when all quartets of taxa in the network satisfy the four-point condition, the network reduces to the phylogenetic tree constructed by the *neighbour-joining* algorithm (Bryant & Moulton, 2004) and the *δ*-score and *Q*-residual are zero.

## Results

We impute missing data using VBPCA, perform dimension reduction and soft-clustering via archetypal analysis, and construct phylogenetic networks using the Neighbor-Net algorithm. The following summarises our results, with details in the supplementary information (SI).

### Cultural transmission in Austronesia

**Figure 3.**
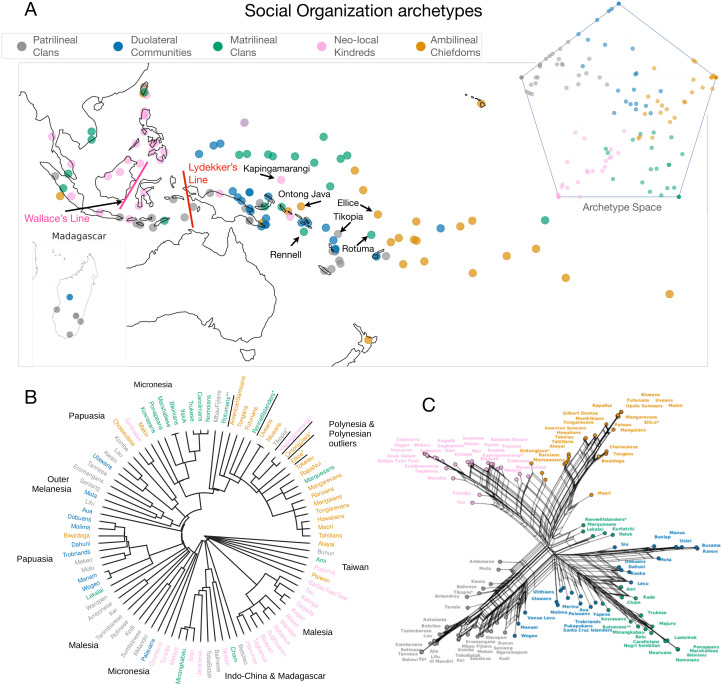
Variation in social organisation. Distribution of Austronesian cultures, coloured by most likely cluster, over (a) geography, (inset) archetypes, (b) Austronesian language tree and (c) the phylogenetic network. The Polynesian outliers in (a) are labelled with text arrows. Lydekker's line and Wallace's line are proposed geographic barriers separating the Asian and Australian biospheres (Ali & Heaney, 2021).

Two pieces of evidence suggest that significant differences in modes of cultural transmission exist among the cultural classes. First, a clear geographic gradient exists in the *cultural interaction* archetypes (Figures S1–S3 and Table S1). Second, cultural amalgamation occurs when cultures combine their influences without one dominating another, as with genetic admixture (Yinger, 1981; Dussart, 2003; Saghar, 2023). There are apparent differences in cultural amalgamation in both the archetype and principal component plots of the *religion* archetypes relative to the *social organisation* and *subsistence* classes (Figures 2, 3 and S3–S6). Together, these results suggest that these cultural classes are determined by distance from the mainland and neighbouring islands and are more subject to macro non-vertical transmission processes (e.g. parallel evolution, exchange, competition; see Gray et al., 2010; Turchin & Gavrilets, 2021) compared with the *social organisation* and *subsistence* classes.

#### Cultural and linguistic outliers

We define cultural outliers as societies whose geographic neighbours are culturally distinct from them, analogous to the definition of *enclaves* by Barbieri et al. (2022). We hypothesised that such outliers can be detected in cultural classes with low degrees of amalgamation but not those with high degrees of amalgamation. To qualitatively test this hypothesis, we utilised known linguistic outliers. *Polynesian outliers* are cultures that speak Polynesian languages but are geographically outside of Polynesia proper, mainly in Micronesia, Papuasia and Outer Melanesia (see Pawley, 1967; Feinberg & Scaglion, 2012; Wilson, 2018). Nine Polynesian outliers were represented in at least one of our datasets (Figures 3 and S3–S5). Rotuma is not considered a Polynesian outlier but is known to have been strongly influenced by Polynesian cultures, with many of its words borrowed from Polynesian languages (Biggs, 2003; Bromham et al., 2015).

We first confirmed that the regions containing Polynesian outliers are distinct from Polynesia in their archetype distributions. For the *cultural interaction* class, the Polynesian archetype distribution is significantly different from that of Papuasia (but not from other regions; see Table S2). For the within-region variability of archetypes among those regions containing Polynesian outliers, Polynesia has the least and second-least intra-regional variance in *social organisation*, and *subsistence*, respectively (Figure S7 and Tables S3–S7). We searched for cultural outliers in these cultural classes by fitting archetype distributions to each region containing Polynesian outliers and then did the same for Polynesia. For a given outlier, we calculated the log-likelihood of its archetypes for both the Polynesian archetype distribution and the archetype distribution for the geographic region of the outlier (see the Methods section and supporting information, SI, for full details). We found that five and four, for the *social organisation* and *subsistence* respectively, out of the six Polynesian outliers in EA are also cultural outliers. For *social organisation*, Ellice, Kapingamarangi, Ontong Java, Rennel and Rotuma are also cultural outliers, but Tikopia is not. We observe that Tikopia is most strongly associated with the patrilineal clans archetype, the dominant archetype in Outer Melanesia (Figure S9). For *subsistence*, only Ellice and Kapingamarang are not outliers, but both are in Micronesia, which has broadly similar dominant subsistence archetypes to Polynesia. In contrast, none of the nine Polynesian outliers in Pulotu were also *religion* outliers, and only one of three Polynesian outliers in Papuasia (Ontong Java) was a *cultural interaction* outlier (Tables S8 and S9).

There is correspondence between cultural and linguistic outliers (Tables s3-s7) in some but not all cultural classes (figures 3 and s3–s5 insets). we hypothesised that the *social organisation* and *subsistence* cultural classes were relatively more vertically transmitted than the *religion* cultural class because these correspondences between cultural and linguistic outliers suggest that there was strong continuity in *social organisation* and *subsistence* traits between subsequently settled islands.

#### Cultural distances and phylogenies

To assess the degree of vertical transmission in each class, we defined a distance between cultures, used it to construct phylogenetic networks and measured the tree-likeness of these networks. We computed the euclidean distance between cultures in the archetype space to account for covariation between cultural traits. Using standard distance metrics such as Jaccard or Hamming directly in the trait space (rather than the archetype space) underestimates the degree of cultural variation (Figures S17–S19). The distribution of cultural distances in the *social organisation* cultural class shows a strong skew, and that of the *subsistence* class is bimodal. In contrast, distances in the *religion* cultural class are approximately normally distributed (Figure 2), resulting in a lower average distance between societies in the *religion* cultural class. These differences in the distributions of cultural distances suggest varying degrees of amalgamation in *religion* across the path of Austronesian expansion, unlike the *social organisation* and *subsistence* cultural classes, where most cultures have a few ‘close’ cultures and many that are more ‘distant’.

We constructed phylogenetic networks for each cultural class by applying the Neighbor-Net algorithm (Bryant & Moulton, 2004) on the cultural pairwise distances (Figures 3, S4 and S5). The *cultural interaction* class was excluded from this analysis because, with only two archetypes, its network is reduced to a tree with two branches corresponding to the two archetypes (Figure S11). Qualitatively, we observe that clustering on the linguistic trees and phylogenetic networks is well described by the archetypes for both the *social organisation* and *subsistence* classes, with few outliers. In contrast, the *religion* class has many more outliers on both the linguistic tree and the cultural phylogenetic network (Figures 3, S4 and S5). We measured the tree-likeness of the cultural phylogenetic networks, i.e. the degree to which processes of vertical transmission are consistent with each network, using *δ*-scores (Holland et al., 2002), which indicate that the networks of the *social organisation* and *subsistence* classes are more tree-like than that of *religion* (Figure 2, Table S9), suggesting that the effect of vertical transmission is stronger in these cultural classes. We focus on the *δ*-score because it is a more accurate measure of reticulation and because, for linguistic data, the *Q*-residual, a related measure (Gray et al., 2010), is highly sensitive to lengths of terminal branches, i.e. the magnitude of pairwise distances in the underlying distance matrix (Holman et al., 2011). The *δ*-score provides consistent results across the choice of pairwise distance (Figure S20).

### Cultural covariation across the Austronesian expansion

Here, we focus on the covariation of cultural archetypes, geography and cultural traits within cultural classes. The SI comprehensively analyses how archetypes are named and their correlations with cultural traits.

#### Covariation between culture and geography

**Figure 4.**
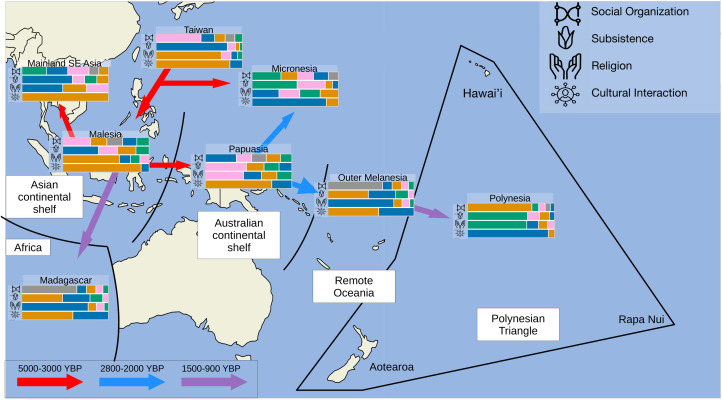
Regional variation in distributions of cultural archetypes. Archetype distributions are given by horizontal-coloured stacked bars, one per cultural class, computed by fitting a Dirichlet distribution to the archetype matrix. Shifts in archetype distributions correspond to the migration pulses and pauses previously described in Gray et al. (2009). Dates in years before present (YBP) of migrations from Chambers and Edinur (2021).

The *Social organisation* archetypes each have regions where they are geographically dominant. Most archetypes appear to have been present from the beginning of the Austronesian expansion, assuming that the snapshot in time represented by the data is reasonably representative of the more distant past (Chambers & Edinur, 2021; Figures 3, 4 and S9). The duolateral communities archetype may have appeared in either Malesia or Papuasia. There is a distinct shift in the dominant archetype between Malesia and Papuasia, Outer Melanesia and Polynesia, which were settled subsequently. This shift is compatible with a pause in Malesia after the initial pulse out of Taiwan (Gray et al., 2009; Figure 4). The combination of matrilineal and patrilineal practices present in the duolateral communities archetype may be at least partially explained by geography: it is the dominant archetype in between geographic regions where the matrilineal (Micronesia) and patrilineal (Outer Melanesia and southeast Malesia) clans archetypes are most common (Figure 3).

For the *subsistence* archetypes, examination of the regional archetype distributions suggests that the grain & cattle archetype is present from the beginning of the Austronesian expansion (Figure 4), in agreement with the archaeological (cf. Stephens et al., 2019) and anthropological literature (Murdock, 1967; Watts et al., 2015b; Kirby et al., 2016). This archetype seems to have been lost in the migration to Papuasia, where none of the cultures belong to this archetype (Figures 4, S4 and S10). The roots & pigs and the fish & fruit archetypes and cultures with a mixture of these archetypes appear in Papuasia. Moreover, Chamorro and Palau in Micronesia are thought to have been settled earlier, with the first Austronesian people arriving approximately 3500 YBP, and then a large wave of settlment from Papuasia around 2200 YBP (Chambers & Edinur, 2021). This settlement pattern may explain the presence of the roots & pigs and agriculture (but not cattle & grain) archetypes on some Micronesian islands.

Among the *religion* archetypes, cultures with the Autonomous Communities archetype separate the *Mana* and Heroes archetypes, which have religious authority that extends beyond the individual community (Figures 4, S5 and S11). The *Mana* and Heroes archetypes are centred around *cultural heroes* and the concept of *Mana* in different forms; both archetypes focus on *deified ancestors* but with varying intensity. In contrast, the primary unifying features of the autonomous communities archetype are the *absence* of super-local religious authority, a focus on *nature spirits* and a *lack* of the importance of deified ancestors (Figure S11). Sheehan et al. (2023) found evidence for the co-evolution of political and religious authority in Austronesian cultures. This may be due to the relatively loose social structure of cultures associated with the autonomous communities archetype, which is positively associated with the duolateral communities archetype, whose social structure is also associated with *acephalous* (‘lacking a head’) *communities* (Figures S11–S13).

#### Covariation between cultural traits: Social Organisation

It is often assumed that lineal biases in residence and kinship reckoning patterns align. Patrilineal and patrilocal practices are broadly present in the duolateral communities and patrilineal clans archetypes. The matrilineal clans archetype shows patterns opposite to the patrilineal clans archetype, with husbands relocating to the wife's kin group and inheritance of land and property by sisters’ sons. These archetypes have uniformly female- and male-biased kinship and inheritance rules, respectively. However, as Fortunato (2019) shows, such alignments of lineal bias do not always occur. Indeed, the Duolateral Communities archetype is associated with a mixture of lineal biases. This archetype has matrilineal *inheritance of land* and *inheritance of moveable properties by sister's sons*, but patrilocal residence patterns.

Further, our results suggest that kinship reckoning and broader community organisation have complex patterns of covariation (Figures 3 and S12). We find that clans are present with opposite lineal biases in two of the archetypes (matrilineal and patrilineal clans) and that cultures with various modes of (some) unbiased inheritance or kinship reckoning run the gamut from autonomous communities (the duolateral communities archetype) to loose neo-local cognatic kindreds (descent through any combination of male and female ancestors, the neo-local kindreds archetype), to stratified chiefdoms with an entrenched elite class (the ambilineal chiefdoms archetype). Possible explanations for variation in modes of social organisation include a ‘bottleneck’ linked to population size, with more stratified social structures developing in societies with larger populations, inheritance from mother cultures and conquest or influence by more stratified societies (Earle, 1997; Feinman and Marcus, 1998; Yoffee, 2005; Marcus, 2008; Rácz et al., 2020; Turchin & Gavrilets, 2021). For results on covariation between cultural traits in the other cultural classes, see the SI.

## Discussion

Four cultural classes were identified: *social organisation*, *subsistence*, *religion* and *cultural interaction*. There are significant differences in the verticality of transmission between the cultural classes; different patterns of variation in kinship residence, inheritance rules and descent reckoning (Figures 3 and S12); support for pulses and pauses in the Austronesian expansion (Figure 4) previously inferred from linguistics (Gray et al., 2009) and genetics (Chambers & Edinur, 2021; Ioannidis et al., 2021); a general west-to-east gradient in the degree of isolation, but also culturally significant exceptions to this gradient (Figures S1–S3, Table S1); and correspondence between linguistic outliers and cultural outliers (Tables S8 and S9).

### Cultural and linguistic outliers

We found that five of six Polynesian outliers – cultures outside of the Polynesian Triangle that speak Polynesian languages – are more similar to Polynesian cultures than to their neighbouring cultures in their profiles of *social organisation* archetypes, and four of six *subsistence* archetypes (Table S8 and S9). Indeed, the ambilineal chiefdoms archetype, common in Polynesia, shows stratified community organisation with high levels of authority (Sheehan et al., 2023), which in turn could have resulted in the transmission of this archetype and associated cultural traits to newly settled communities.

In contrast, in the *religion* cultural class, none of the nine Polynesian outliers in Pulotu were cultural outliers; similarly, in the *cultural interaction* class, only one of three Polynesian outliers in Papuasia (Ontong Java) is an archetype outlier (Table S9). For the *religion* cultural class, this may be explained by greater cultural amalgamation relative to the *social organisation* and *subsistence* cultural classes (Figure 2). In the *cultural interaction* cultural class, it may be explained by geography: the distance between islands in Polynesia and the regions containing Polynesian outliers is relatively greater than in other regions of Oceania (Figures S1–S3).

### The path of Austronesian expansion

Lydekker's line and Wallace's line are proposed biogeographic barriers separating the Asian and Australian biospheres (Ali & Heaney, 2021, and Figures 3 and S3–S5). The channel between the islands of Borneo and Celebes delineates Wallace's line. Lydekker's line corresponds to the western end of the Australian continental shelf. In the *subsistence* cultural class, we found a striking shift in the archetype distribution away from the grain & cattle archetype (Figure S4), which seems to agree with the previously inferred pause in the Austronesian expansion between Malesia and Papuasia (Gray et al., 2009). At the last glacial maximum, when much of Malesia, but not Australia, was connected to mainland Asia, the Banteng (*Bos javanicus*, a species of wild bovine) reached the eastern end of the Asian continental shelf (Ju et al., 2007; Gardner et al., 2014). Genetic evidence also indicates that Indonesian varieties of cattle were domesticated locally (Chapter 8 of Bellwood, 1995), which may explain why Lydekker's line (but not Wallace's line) appears to delineate the presence and absence of cattle. Some islands probably could not support cattle (e.g. small islands off the coast of Papua New Guinea). However, there are islands in Polynesia that, in the post-colonial context, are associated with large-scale animal husbandry: Aotearoa (New Zealand) famously has more sheep than people (Morris, 2009), and most of the modern agricultural land use in Hawai'i is dedicated to livestock (Rehkamp et al., 2021). However, in the pre-colonial context, Polynesia is associated with the roots & pigs and fish & fruit archetypes. The lack of widespread animal husbandry in Polynesia may be attributed to the loss of the grain & cattle and agriculture archetypes during the Austronesian expansion before the arrival to Polynesia. This lack of pre-colonial large-scale husbandry, combined with evidence for local domestication and the distribution of wild bovines, suggests that cattle were not transported with voyagers on the path of expansion. The accidental loss of valuable plants and animals during sea voyages (Whistler, 2009; Wilme et al., 2016) may also have played a role in shifting away from grain & cattle.

Patterns of variation in the four *religion* archetypes are consistent with previous analyses of the Pulotu dataset that found evidence for the co-evolution of religious and political authority (Sheehan et al., 2023). Notably, Polynesia is associated with *super-local religious authority* and *dual stratification into a hereditary aristocracy and a lower class* (Figures S11 and S12). Furthermore, the presence of all *religion* archetypes in Malesia and regions further east (Figures S5 and S15) and the finding that Polynesian outliers are not *religion* outliers (Table S9) suggest that the evolution of the *religion* cultural class was strongly affected by non-vertical mechanisms such as parallel evolution or cultural exchange resulting in religious syncretism.

We found a west-to-east gradient of increasing Isolation (Figures S1–S3 and Table S1), with notable exceptions. In Polynesia, Tonga is in the contact archetype (Figure S3), which may be due to its period of expansion during the Tu'i Tonga Empire (Mageo, 2002). Similarly, Rapa Nui, Samoa, Mahoi and Tokelau have some degree of contact (although less than isolation, Figure S2). Among these, Samoa and Tokelau are known to have fallen within the Tongan sphere of influence (Gunson, 1990; Geraghty, 1994) and Rapa Nui has been suggested to have been in contact with South American cultures (Scaglion, 2005; Chambers & Edinur, 2021; Ioannidis et al., 2021). In Micronesia, Ulithi is primarily associated with the contact archetype, which, together with the presence of pre-Austronesian people, may be explained by its position in western Micronesia (Watts et al., 2015b). In Papuasia, three cultures are more associated with isolation than contact: Ontong Java, Rennell and Bellona, all of which have Polynesian languages.

### Covariation of cultural traits

We found five archetypes in the *social organisation* cultural class (Figure 3). The matrilineal and patrilineal clans archetypes appear to have a predominantly female (matrilineal) or male (patrilineal) bias in residence, inheritance, and descent reckoning. In contrast, the neo-local kindreds archetype shows little, if any, lineal bias (Figure S12). Furthermore, the duolateral communities archetype shows a female bias in inheritance, a male bias in residence, and unbiased (duolateral) descent reckoning. This mixture of kinship practices may be explained by the geographic distribution of this archetype, located between regions in which patrilineal practices are common to the west and east and matrilineal practices to the north (Figure 3c). In addition, the ambilineal chiefdoms archetype shows unbiased (ambilineal) descent reckoning and male-biased land inheritance, which further support a ‘shift in focus – from viewing matrilineal and patrilineal kinship as unitary phenomena to consideration of the different aspects of the social system featuring a bias towards lineally related kin’ (Fortunato, 2019), and a more general shift away from ‘main sequence theory’, as suggested by Opie et al. (2014). Genetic evidence and cultural phylogenetic analyses indicate that early Austronesian societies were matrilocal (Jordan et al., 2009; Fortunato & Jordan, 2010). Thus, the persistence of matrilocal practice in Micronesia may be (partially) explained by the early settlement of Chamorro and Palau early in the Austronesian expansion and its relative isolation (Figure 4).

It is possible to have more clusters than archetypes, for example, in population genetics and genomics, when multiple individuals have similar levels of admixture (Behr et al., 2016). Similarly, some cultural groups may have similar archetype mixtures. In the *subsistence* cultural class, we found four archetypes but five clusters. The fifth cluster is a mixture of the roots & pigs and fish & fruit archetypes. Geographically, fish & fruit, roots & pigs and their mixture dominate in all regions except for Island Southeast Asia and Madagascar, where grain & cattle dominates (Figures S4 and S10).

In the anthropological and archeological literature, primarily based on Eurasian cultures, it has often been assumed that grain-based agriculture (e.g. maize in the Americas, wheat in the Levant and rice in East Asia) is a pre-requisite for the rise of a complex society (complex meaning chiefdoms and states; see Moseley & Feldman, 1988; Earle, 1997; Feinman & Marcus, 1998; Yoffee, 2005; Marcus, 2008; Beresford-Jones et al., 2018). The Austronesian chiefdoms represent a challenge to the necessity of *grain-based* agriculture as the primary mode of subsistence for the rise of complex societies owing to (i) the absence of correlation between the Ambilineal Chiefdoms archetype and *grain agriculture*, (ii) these chiefdoms’ lack of association with metalworking and pottery production and (iii) their association with the fish & fruit and roots & pigs archetypes (Figures S11 and S12). These findings are further backed by evidence for widespread sweet potato cultivation in the archaeological records of Polynesia, but no evidence for grain cultivation (Kirch et al., 2004; Kirch, 2017).

Additional evidence from the Americas suggests that only a sufficiently stable and stationary food source and, sometimes, appropriate local trade partners are required for complex societies to rise. For example, complex societies in the modern Pacific Northwest are thought to have relied on socio-ecological systems and the rich local biodiversity for subsistence (Trosper, 2003). The most appropriate analogy for the Polynesian chiefdoms in challenging the necessity of grain-based agriculture may be the complex societies on the northwest coast of South America. Andean archaeologists have developed the *Maritime Foundations of Andean Civilisation* hypothesis, which directly challenges the notion that grain-based agriculture is necessary for the rise of complex societies (Moseley, 1975, 1992, 2005; Moseley & Feldman, 1988; but see Beresford-Jones et al., 2018). On the arid coastal plane of Peru, which has the world's richest fishery immediately off-shore, the rise of chiefdoms and states (e.g. Caral–Supe) occurred only in the north, where there were inland trade partners with the plant fibres necessary to make the nets needed to catch large amounts of fish (Beresford-Jones et al., 2018). Thus, both the Polynesian chiefdoms and the complex societies of the northern Peruvian coast represent an alternative model in which it is *not* necessary for the materials needed for the acquisition and storage of food to be locally produced, and where grain-based agriculture is not required if suitable trade partners and sufficiently rich sources of subsistence are available.

We note that Sheehan et al. (2018) found support for coevolution between landesque capital intensive agriculture and political complexity, which appears to contrast with our findings. This difference is probably due to the different operationalisation of variables, with Sheehan et al. (2018) focussed on intensive agriculture and a high level of political complexity rather than just the presence of agriculture and hereditary chiefs.

The high degree of religious and political authority, together with the relative geographic isolation of each culture in Polynesia, may explain why it has the most within-region variation in *religion* archetypes, despite its position at the terminal end of the Austronesian expansion (Figures S7). Specifically, the relatively greater geographic distance between neighbours and the high degree of authority may combine to preserve differences among individual cultures. When paired with occasional contact with other cultures, this greater consolidation of power may result in greater variation owing to fewer amalgamation events from trade and other contact.

### Modes of cultural transmission

We inferred that the *social organisation* and *subsistence* cultural classes are relatively more vertically transmitted than the *religion* cultural class (Figure 2 and Table S10). This is supported by measurements of the tree-likeness of the phylogenies of each cultural class, the distributions of pairwise archetype distances (Figure 2), and the degree of separation and mixing between individual cultures and archetypes in principal components spaces, linguistic trees and cultural phylogenetic networks (Figures 3, S4, S5 and S8). These results are relative, and none of the metrics considered are absolute measures of vertical transmission (Holland et al., 2002; Gray et al., 2010).

At the macroevolutionary scale one might *a priori* expect *religion* to be relatively more vertically transmitted then *social organisation* or *subsistence*. Cultural group selection (Smith, 2020) may drive the adoption and spread of religious beliefs, and these beliefs may be maintained by mechanisms such as costly sacrifice (Atran & Henrich, 2010; Norenzayan, 2013; Norenzayan et al., 2016). However, the relatively lesser degree of verticality in transmission of religious traits may be explained by the interplay of both intra- and intercultural factors. Vlerick (2020) argues that many features of religious practice are not generated by top-down control, but rather from the bottom up. Thus one possible explanation for the relatively lower verticality of the *religion* class is the combination of various modes of cultural exchange and tension between top-down religious authority (i.e. super-local religious structures; Sheehan et al., 2023) and bottom-up adoption of different beliefs and practices.

However, examination of the individual traits within the religion class suggests that some other factors are at play in the transmission of religious traits. When we checked for phylogenetic signal of individual traits over the linguistic phylogeny of Gray et al. (2009), we found that while some traits had strong phylogenic signal (i.e. stronger verticality, e.g. headhunting with *λ* = 0.52, *p* = 4 × 10^−5^.) and others had none (e.g. cultural heroes with *λ* = 0.034 *p* = 0.7; see Table S13 for a complete list), the majority were somewhere in between.

Both vertical and non-vertical transmission, as well as other factors such as adaptation, probably played a role in cultural evolution in Austronesia, as even the relatively more tree-like cultural classes are only moderately tree-like (Holland et al., 2002; Gray et al., 2010). In agreement with previous studies (Gray et al., 2010), our results suggest strong non-vertical cultural transmission in Polynesia and throughout the Austronesian expansion. A possible explanation for this non-vertical transmission is the advanced maritime technology of Austronesian societies (Spriggs, 1995; Bellwood et al., 2011; Kirch, 2017). Societies in the contact archetype are associated with frequent contact (Figure S16). We also detect a geographic gradient in isolation (Figures 4 and S1–S3), which suggests that the degree of contact vs. isolation drives cultural evolutionary processes rather than being an outcome of such processes. Essentially, the degree of isolation, while potentially mitigated by the maritime technologies that allowed Austronesians to colonise islands previously uninhabited by humans, appears to be primarily explained by geography and ecology (Kirch, 2017). For example, the ability of Rapa Nuians to maintain their canoes upon arrival may have been hampered by the eventual loss of trees on the island (see Hunt & Lipo, 2013), as well as their relative geographic isolation. Similarly, different ‘canoe plants’ and ‘canoe animals’, taken with voyagers settling new islands, were lost at various stages of the Austronesian expansion (Whistler, 2009; Wilme et al., 2016), which may be explained by some plants and animals or the knowledge required to sustain them not surviving subsequent voyages. These results align with those of Padilla-Iglesias et al. (2020), who found that geographic and cultural isolation are drivers of linguistic evolution in Austronesian cultures.

Many studies have applied models of character evolution over phylogenetic trees to study the evolution and co-evolution of cultural traits over linguistic phylogenetic trees (Holden & Mace, 2003; Mace & Holden, 2005; Jordan et al., 2009; Fortunato & Jordan, 2010; Opie et al., 2014; Watts et al., 2015a, 2016; Sheehan et al., 2018, 2023; Surowiec et al., 2019). However, concerns have been raised about the appropriate application of such approaches to cultural evolution (Evans et al., 2021). Indeed, non-vertical transmission via macro-cultural processes such as innovation, conquest, exchange and competition may result in lower degrees of cultural diversity owing to cultural amalgamation and assimilation (Dyson-Hudson & Smith, 1978; Marcus, 2008; Karin & Alon, 2018; Yeh et al., 2019; Turchin & Gavrilets, 2021; and Figures S5 and S15). For Austronesian societies in particular, we observe that cultural amalgamation results in lower average cultural distances in the *Religion* class (Figure 2). Further, using traditional pairwise distances in the trait space without accounting for linkage may underestimate cultural variation (Figures S17–S19). Finally, failure to account for linkages between cultural traits and degree of vertical transmission may also introduce biases and errors into such analyses (Yeh et al., 2019; Evans et al., 2021), similar to the biases introduced by genetic admixture to inferences of population history from genetic data (Royal et al., 2010). Consistent with the findings of Holman et al. (2011) for linguistic data, we also find that relative values of the *Q*-residual (Gray et al., 2010) are sensitive to the choice of pairwise distance for cultural trait data. Thus, our results call for further research on non-vertical cultural transmission and the application of statistical methods used in population-genetic analyses to cultural evolution.

## Supporting information

Macdonald et al. supplementary material Macdonald et al. supplementary material

## Data Availability

The raw input data from EA and Pulotu is available through D-place (Kirby et al., 2016, https://d-place.org). All output data produced by this work are available in Zenodo along with the software files (Macdonald et al., 2024a).

## Supplementary material

The supplementary material for this article can be found at https://doi.org/10.1017/ehs.2024.45.

## Transparency and reproducibility

The authors have made the code used to produce these results available in the Zenodo repository (Macdonald et al., 2024a).
