# Supplementary material for: Cultural transmission, networks, and clusters among Austronesian-speaking peoples
Source: Evol Hum Sci. 2024 Dec 6;6:e51. doi: 10.1017/ehs.2024.45 (PMC11658950; doi:10.1017/ehs.2024.45)
Supplement: Macdonald et al. supplementary material [file S2513843X24000458sup001.pdf]

Supplementary Information for Cultural transmission, networks, and clusters among Austronesian-speaking peoples

Joshua C. Macdonald[1], Javier Blanco-Portillo[2], Marcus W. Feldman[2,*], and Yoav Ram[1,3,*]

[1]School of Zoology, Faculty of Life Sciences, Tel Aviv University, Tel Aviv, Israel
[2]Department of Biology, Stanford University, Stanford, CA, USA
[3]Sagol School of Neuroscience, Tel Aviv University, Tel Aviv, Israel
[*]Corresponding author, MWF: mfeldman@stanford.edu, YR: yoavram@tauex.tau.ac.il

July 17, 2024

Contents

S1 Supplementary Results **1**

S1.1 Imputation 1

S1.2 Denoising 1

S1.3 Effect of missing data on downstream analysis 2

S1.4 Naming of archetypes 2

S1.5 Cultural cluster analysis 3

S1.6 Hierarchical clustering 4

S2 Supplementary tables and figures **5**

List of Figures

S1 **Regional distance comparison to nearest neighboring culture.** A value of 0 indicates that all regional cultures have an immediate neighbor. 5

S2 **Cultural Interaction bar-plots with respect to archetypes**. Cultures are separated by geographic region. 6

S3 **Variation in Cultural Interaction**. Over **(a)** geography, **(inset)** archetypes (the Polynesian outliers in this data are labeled with text arrows), and **(b)** Austronesian language tree. Lydekker's line and Wallace's line are proposed geographic barriers separating the Asian and Australian biospheres (see Ali and Heaney, 2021). 7

S4 **Variation in Subsistence**. Austronesian cultures, colored by most likely cluster, over **(a)** geography, **(inset)** archetypes (the Polynesian outliers in this data are labeled with text arrows), and **(b)** Austronesian language tree. Lydekker's line and Wallace's line are proposed geographic barriers separating the Asian and Australian biospheres (see Ali and Heaney, 2021). 8

S5 **Variation in Religion**. Austronesian cultures, colored by most likely cluster, over **(a)** geography, **(inset)** archetypes (the Polynesian outliers in this data are labeled with text arrows), and **(b)** Austronesian language tree. Lydekker's line and Wallace's line are proposed geographic barriers separating the Asian and Australian biospheres (see Ali and Heaney, 2021). 9

S6 **Two-dimensional PCA projections of the cultural data**. **(A)** *Social Organization* data; loadings are: I) Token bride price, II) Polygamy, III) Significant bride price prevailing pattern, IV) Bride service, V) Domestic organization: small extended families, VI) Significant bride price, alternative pattern. Thus, PC 1 captures differences in domestic organization and PC 2 differences in transactions/exchange at marriage. **(B)** *Subsistence* data; loadings are I) Animal husbandry 46-55% of subsistence, II) Fishing 16-25% of subsistence, III) Fishing 6-15% of subsistence, IV) Agriculture 0-5% of subsistence, V) Complete absence of agriculture, VI) Fishing 36-45% of subsistence. Thus, PC 1 captures differences in the importance of animal husbandry and fishing and PC 2 differences in the importance of agriculture. **(C)** *Religion* data; loadings are I)Actions of others after one has died do not affect the nature of one's afterlife, II) No myth of man's creation, III) Belief in cultural heroes is a major focus, IV) Belief in nature spirit(s) a major focus, V) Creationist myth of humans, VI) Actions while living are the principal determinant of the nature of one's afterlife. Thus, PC 1 captures differences in religious focus, and PC 2 differences in creation myths. **(D)** *Cultural Interaction* data; loadings marked by I) Pre-Austronesians absent, II) Common conflict between communities, III) Occasional conflict between communities, IV) Frequent conflict with other societies, V) Pre-Austronesians present, VI) Common conflict with other societies. Thus, PC 1 captures the presence/absence of pre-existing populations at the time of settlement, and PC 2 captures differences in the nature and frequency of conflict within and between societies. 10

S7 **Within geographic region variability of archetype sets.** Cumulative density plots for F_{st}/F_{st}^{max}, which is proportional to Dirichlet distribution variance in Polynesia and regions containing Polynesian outliers. 11

S8 **Cultural Interaction network**. Due to the binary nature of these archetypes, the network reduces to a tree with only two branches. 11

S9 **Social Organization bar-plots with respect to archetypes**. Cultures are separated by geographic region. 12

S10 **Subsistence bar-plots with respect to archetypes**. Cultures are separated by geographic region. 13

S11 **Binarized Religion trait correlations with archetypes.** Only features (n = 89) that have correlation P-values less than 0.001 after FDR correction are displayed (n = 47). 14

S12 **Binarized Social Organization trait correlations with archetypes.** Only features (n = 236) that have correlation P-values less than 0.001 after FDR correction are displayed (n = 87). 15

S13 Hierarchical cluster-map on the Spearman correlation matrix for both archetypes and non-cultural features across the 65 cultures in common between EA and Pulotu. There are three clusters that appear to correspond to the results of Karin and Alon (2018). . 16

S14 **Binarized Subsistence trait correlations with archetypes.**. Only features ($n = 121$) that have correlation P-values less than 0.001 after FDR correction are displayed ($n = 76$). Note that the Agriculture archetype is missing many features on average (Table S11) and is primarily associated with (1) this missigness and (2) agriculture of unspecified type providing most of subsistence. 17

S15 **Religion bar-plots with respect to archetypes**. Cultures are separated by geographic region. 18

S16 **Binarized Cultural Interaction trait correlations with archetypes.** Only features ($n = 18$) that have correlation P-values less than 0.001 after FDR correction are displayed ($n = 10$). 19

S17 **Kmeans clustering metrics for distances calculated in archetype space**. Optimal numbers of Kmeans clusters for each data class (rows) according to the silhouette score (column 1), the variance ratio criterion (column 2), and the Davies-Bouldin score (column 3). For all archetypes except *Subsistence*, the optimal number of clusters on the archetypes is the same as the number of archetypes. 20

S18 **Kmeans clustering metrics for Hamming distances**. Optimal numbers of Kmeans clusters for each data class (rows) according to the silhouette score (column 1), the variance ratio criterion (column 2), and the Davies-Bouldin score (column 3). The Hamming distance underestimates the number of clusters. 21

S19 **Kmeans clustering metrics for Jaccard distances**. Optimal numbers of Kmeans clusters for each data class (rows) according to the silhouette score (column 1), the variance ratio criterion (column 2), and the Davies-Bouldin score (column 3). The Jaccard distance underestimates the number of clusters. 22

S20 **Comparison of δ-score and Q-residuals**. The δ-score preserves relative differences in tree-likeness across choice of pairwise distance, but the Q-residual does not. . . . 23

S21 **Imputation performance comparison.** Accuracy of VBPCA imputation relative to other imputation algorithms across 50 replicates, each with 2% values deleted randomly from one of the four cultural class datasets. Given that none of the imputers consistently outperforms VBPCA and that VBPCA provides marginal posterior estimates for the data matrix (which other imputers do not), VBPCA is used for the remainder of our analysis. Positive values indicate that VBPCA performs better. The vertical lines are 95% confidence intervals for the difference in accuracy. The alternative imputers are Iterative imputer with one of the following regressors: Bayesian ridge regressor (IIBR), Iterative imputer with decision tree regressor (IIDT), Iterative imputer with nearest neighbor regressor with $k = 5$ (IIKNN5) and $k = 10$ (IIKNN10), Iterative imputer random forest regressor (IIRF), and simple imputation with missing data replaced by column means (Simple). 24

S22 **VBPCA noise filtering for different datasets.** The proportion of cumulative variance explained by the optimal number of principal components for data reconstruction as determined by VBPCA using classical PCA after filling missing values via different imputation approaches. 25
S23 **The relationship between Accuracy and MSE**. Across all datasets and imputers, there is a strong correlation between MSE (mean squared error) and the accuracy of the inferred values. 25
S24 **Cost functions for each of the four cultural classes**. The colors of the lines get darker as the number of principal components increases, and the optimal number of PCs (49, 37, 34, 13 for each of the datasets) is colored in black. As the optimum is approached, the values of the cost function for a given number of principal components begin to 'stack up.' 26

List of Tables

S1 Distance to nearest neighbor (km) by geographic region. A value of 0 indicates immediate adjacency to the nearest neighbor. 27
S2 Likelihood ratio test for regional archetype distributions for geographic regions containing Polynesian outliers and Polynesia. 27
S3 Pairwise comparisons of F_{st}/F_{st}^{max} for *Social Organization* archetypes using Wilcoxon rank sum test with continuity correction. 27
S4 Pairwise comparisons of F_{st}/F_{st}^{max} for *Subsistence* archetypes using Wilcoxon rank sum test with continuity correction. 27
S5 Pairwise comparisons of F_{st}/F_{st}^{max} for *Religion* archetypes using Wilcoxon rank sum test with continuity correction. 28
S6 Pairwise comparisons of F_{st}/F_{st}^{max} for *Cultural Interaction* archetypes using Wilcoxon rank sum test with continuity correction. 28
S7 Normalized F_{st} values for regions containing Polynesian outliers and Polynesia itself. 28
S8 Mann-Whitney U test, the difference in log-likelihood; alternative hypothesis Polynesian likelihood is greater. * indicates statistical significance. 28
S9 Mann-Whitney U test, the difference in log-likelihood; alternative hypothesis Polynesian likelihood is greater. * indicates statistical significance. 29
S10 Differences in relative verticality of transmission among cultural archetype sets as indicated by Dunn's test for pairwise differences with Holm's correction for the p-values. 29
S11 Comparison of proportion of missing data within different archetypes and for linguistic outliers. Alone among archetypes, cultures associated with the Agriculture archetype are missing large numbers of features. 30
S12 Correlation of missingness with extreme archetype values. In general the degree of missigness does not have a strong effect on the max/min for a given society and cultural class. 30

S13 *Religion* traits sorted by phylogenic signal over the linguistic phylogeny from Gray et al. (2009). P-values are from LRT with null assumption $\lambda = 0$. 31

S1 Supplementary Results

S1.1 Imputation

Iterative imputer. Iterative imputation works from least to most missing values per column using the imputed values in successive iterations. At each iteration, one feature (cultural trait) is chosen as the dependent variable y, and the other features are treated as independent variables, X. Then features y are iterated according to which of them has the least missing values, assuming the inferred values from the previous iteration. A regression model is fitted to the (X, y) pairs without missing values. The fitted regression model is then used to predict missing y values, ending the current iteration. At the next iteration, another feature is chosen as y, a new regression model is fitted and used to predict missing values, and so on, until all missing features are assigned an expected value. We used the `IterativeImputer` implementation in *Scikit-learn* (Pedregosa et al., 2011), a Python machine learning library. For the initialization step, where a first estimate is obtained, missing values were replaced by the column mean. The following regression models were considered: Bayesian ridge regression (IIBR), nearest neighbors regression with five or ten neighbors (IIKNN5, IIKNN10), decision tree regression (IIDT), and random forest regression (IIRF).

Comparison of imputation strategies. There was a strong correlation between MSE (mean squared error) and accuracy across all datasets and imputation approaches (see Figure S23). Thus, we focused on differences in effect size imposed by differing MSEs and generally found small differences in the accuracy of VBPCA imputation relative to other imputation algorithms across the 50 replicates and the four Austronesian datasets. None of the imputers consistently outperformed VBPCA. Across datasets IIBR was 3.9% less accurate (95% CI 1.1 – 3.8%, *Religion*) and 2.1% (95% CI 1.5 – 2.8%; *Social Organization*) more accurate than VBPCA. IIDT was between 11.8% (9.5-14.1%, *Cultural Interaction*) less accurate and 0.5% (0.01–1.0%) more accurate than VBPCA. IIKNN5 between 3.3% (0.5–6.1%, *Cultural Interaction*) less and 2.3% (0.8–3.8%, *Religion*) more accurate than VBPCA. IIKNN10 between 4.7% (2.2–7.1%, *Cultural Interaction*) less and 4.0% (2.6–5.4%, *Religion*) more accurate. IIRF between 11.5% (9.1-13.9%, *Cultural Interaction*) less and 1.4% (0.9-1.9%, *Social Organization*) more accurate. Simple (replacement of missing values by column means) between 3.4% (2.9-3.8%, *Social Organization*) less and 2.3% (1.0-3.7%, *Religion*) more accurate (see Figure S21).

S1.2 Denoising

The proportion of cumulative variance explained by the optimal number of principal components for data reconstruction as determined by VBPCA using classical PCA after filling missing values via different imputation approaches is a measure of the degree of noise VBPCA filters for each dataset. As can be seen in Figure S22, the *Social Organization* dataset has the most noise filtered out, followed by *Religion*, *Subsistence*, and finally by *Cultural Interaction*. The degree of variance explained by each of the considered imputation strategies is tightly clustered by the dataset and the proportion of noise filtered is not solely a function of the number of significant components, the size of the original data, or the number of PCs for the optimal reconstruction (see Figure S22, and Anonymous, 2023).

S1.3 Effect of missing data on downstream analysis

We checked for differences in the degree of missingness between linguistic outlier and non-outlier cultures in each cultural class and correlations between how much data was missing and archetype loadings. We found no significant differences in the proportion of missing data, except for the Agriculture archetype, between outliers and non-outliers in any of the cultural classes using Dunn's test for stochastic dominance with null hypotheses that there was no difference in stochastic dominance (Table S11).

We also checked for correlation between extreme (max and min) archetype values for each culture and each cultural class and did not find significant correlations between extreme values and the degree of missingness (Table S12).

S1.4 Naming of archetypes

Social Organization. Analysis of the *Social organization* class of variables revealed five cultural archetypes, which we name Duolateral Communities, Patrilineal Clans, Neo-local Kindreds, Matrilineal Clans, and Ambilineal Chiefdoms, based on their correlation with the original variables in this class (Figure S12). Ambilineality is associated with chiefdoms because cultures belonging to this archetype were also associated with *social stratification into a hereditary aristocracy and a lower class* (Spearman correlation, $\rho = 0.45, p < 0.001$), which is a classic characteristic of chiefdoms (Earle, 1997; Feinman and Marcus, 1998; Yoffee, 2005). Duolateral indicates the presence of both matrilineal and patrilineal kin groups; bilateral indicates the absence of matri/patrilineal kin groups and the presence of cognatic kin groups; ambilineal means kin reckoning of both matrilineal and patrilineal kin without set order (Kirby et al., 2016; Fortunato, 2019).

Subsistence. We found four cultural archetypes for the *Subsistence* cultural class, which we name Roots & Pigs, Grains & Cattle, and Fish & Fruits (Figure S14). In addition to modes of subsistence for which the archetypes are named, there were significant differences in labor practices among archetypes. Grains & Cattle archetype is associated with the importance of weaving, pottery-making, and metal-working: this archetype is positively associated with *weaving by most adult females* ($\rho = 0.66, p < 0.001$); *metal-working as a craft specialty* ($\rho = 0.74, p < 0.001$), which is *male-dominated* ($\rho = 0.74, p < 0.001$); and *pottery-making by most adults* ($\rho = 0.61, p < 0.001$). The Grain & Cattle is also notable for being the only archetype with a *presence of slavery in the past* ($\rho = 0.46, p < 0.001$). In contrast, the Roots & Pigs and Fish & Fruit archetypes are both associated with the *lack of importance of metal-working* ($\rho = 0.55, p < 0.001$ for both); the Fish & Fruit archetype is associated with the *lack of importance of pottery-making* ($\rho = 0.43, 0.42$ respectively $p < 0.001$ for both) and the Roots & PIgs and Fish & Fruit are associated with having *never practiced slavery* ($\rho = 0.38, 0.39, p < 0.001$ for both). Finally, alone among the archetypes, Agriculture is associated with a large degree of missing data and the majority of subsistence coming from agriculture of unspecified type.

Religion. We found four archetypes for the *Religion* class, which we name Heroes, *Mana*, Autonomous Communities, and Animism (Figure S11). The Heroes archetype is also associated with the *absence of god(s)* ($\rho = 0.35, p < 0.001$), *combined political & religious authority* ($\rho = 0.47, p < 0.001$), a

religious community composed of the entire society ($\rho = 0.4, p < 0.001$). *Mana* is associated with *super-local (multiple-community) religious authority* ($\rho = 0.51, p < 0.001$), and *social hierarchy tapu* (a *tapu* is a spritual restriction; $\rho = 0.37, p < 0.001$). Finally, the Autonomous Communities archetype is associated with the *absence of religious authority* ($\rho = 0.53, p < 0.001$) and *piercing as a rite* ($\rho = 0.43, p < 0.001$).

Cultural Interaction. Two archetypes, which we name Isolation and Contact, were found in the *Cultural Interaction* cultural class (Figure S16). Because there are only two archetypes, the correlations for isolation and contact are opposite. Contact is positively associated with *the presence of pre-Austronesian populations* ($\rho = 0.85, p < 0.001$), *occasional conflict within* and *between communities* ($\rho = 0.45, p < 0.001$ for both), and *frequent* or *high levels of warfare with other societies* ($\rho = 0.67, 0.52, p < 0.001$ for both). Isolation is positively associated with *occasional contact* ($\rho = 0.49, p < 0.001$), both *frequent* and *moderate conflict within communities* ($\rho = 0.73, 0.46, p < 0.001$ for both), and *rare warfare with other societies* ($\rho = 0.82, p < 0.001$).

S1.5 Cultural cluster analysis

Subsistence. In some circumstances the raw materials needed to produce a good may need to be locally available. However, the lack of local resources to produce an item does not necessarily indicate its absence in a society. The Roots & Pigs and Fish & Fruit archetypes are associated with *lack of importance of metalworking*; the Fish & Fruit archetypes are associated with *lack of importance of pottery-making*. This lack of manufacture does not indicate a lack of use. Large amounts of pottery exist in the archaeological records of islands associated with the Fish & Fruit and Roots & Pigs archetypes (Spriggs, 1995). Genetic variation in "canoe plants" and "canoe animals" - the corpus of plants and animals taken in canoes along the path of migration - together with occasional contact even for cultures belonging to the *Isolation* archetype suggests the presence of trade networks between islands (Whistler, 2009; Wilme et al., 2016). Thus, cultures belonging to the Fish & Fruit, and Roots & Pigs archetypes may have traded locally produced products for pots and metal goods.

Cultural Interaction. Isolation is most common in Micronesia, Outer Melanesia, and Polynesia, likely due to the greater distances between neighbors in these regions (Micronesia 236.5 ± 46.1 km, Outer Melanesia 56.5 ± 15.7 km, Polynesia 747.7 ± 220.7 km (mean ± standard error); Figure S1-S3, and Table S1). Beyond *frequency of interaction with other societies*, the Contact archetype is positively associated with *the presence of pre-Austronesian populations*, *occasional conflict within* and *between communities*, and *frequent* or *high levels of warfare with other societies*. The results further indicate that variation in degree of isolation is primarily driven by distance from neighbors and the mainland.

Isolation is positively associated with both *frequent* and *moderate conflict within communities*, and *rare warfare with other societies*. These results suggest outwardly and inwardly driven conflict, respectively: the primary source of conflict in societies belonging to the Isolation archetype is at the inter- and intra- community levels, whereas societies in the contact archetype appear more focused on conflict with neighbors. Potential explanations for this difference include opportunity (closer proximity

to neighbors, see Figures S7 to S9, Table S1 and González-Ruibal, 2019) and the presence of near neighbors acting as an internally unifying force and target for conflict (González-Ruibal, 2019).

S1.6 Hierarchical clustering

We performed agglomerative hierarchical clustering on the archetypes combined with non-cultural features such as island type, size, and elevation to identify clusters across cultural classes, geography, and ecology (Figure S18). This analysis reveals three clusters for the 65 cultures that are common between EA and Pulotu. Notably, two of these clusters correspond well to the “Resource Competition" and “Resource Defense" clusters described by (Karin and Alon, 2018, Figure S18). Resource competition correlates with Coral Atolls, *Mana*, Isolation, and Fish & Fruit, and geographically corresponds to the Polynesian islands. Resource defense is geographically associated with Melanesia, and is correlated with Contact, larger islands, and Grain & Cattle (Figure S18). These findings indicate that the “ecotypes" suggested by Karin and Alon (2018) are robust to inference method. However, our results also indicate that care must be taken when collapsing cultural data into fewer features. In effect performing hierarchical clustering on the combined archetypes amounts to iterative dimension reduction. Such approaches may remove meaningful cultural variation (for example finding three hierarchical clusters on the combined data versus finding a total of 15 archetypes across four cultural classes).

S2 Supplementary tables and figures

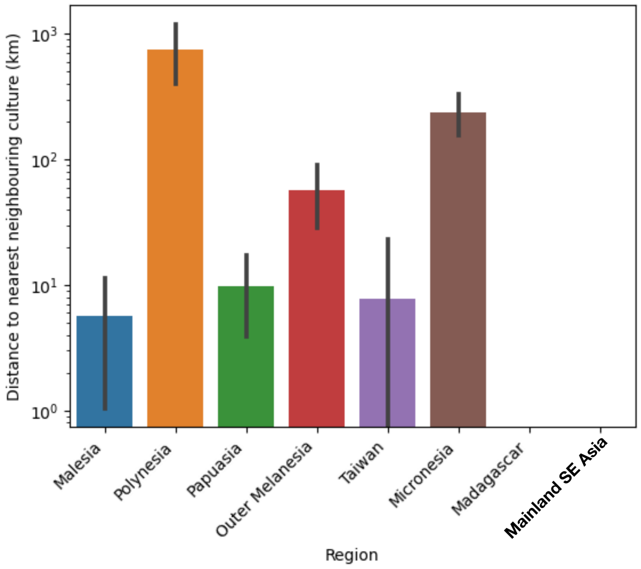

Figure S1: Regional distance comparison to nearest neighboring culture. A value of 0 indicates that all regional cultures have an immediate neighbor.

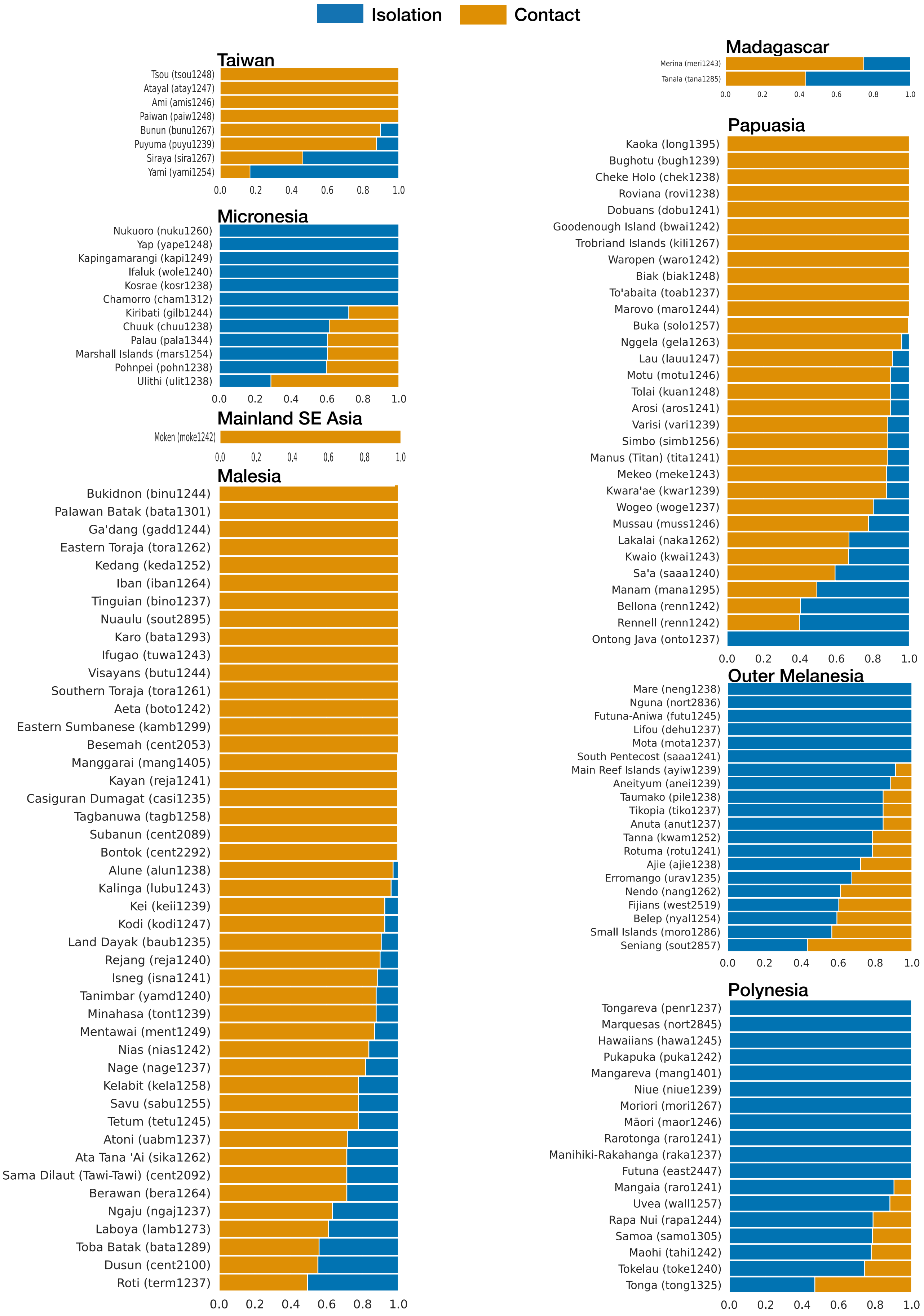

Figure S2: Cultural Interaction bar-plots with respect to archetypes. Cultures are separated by geographic region.

A

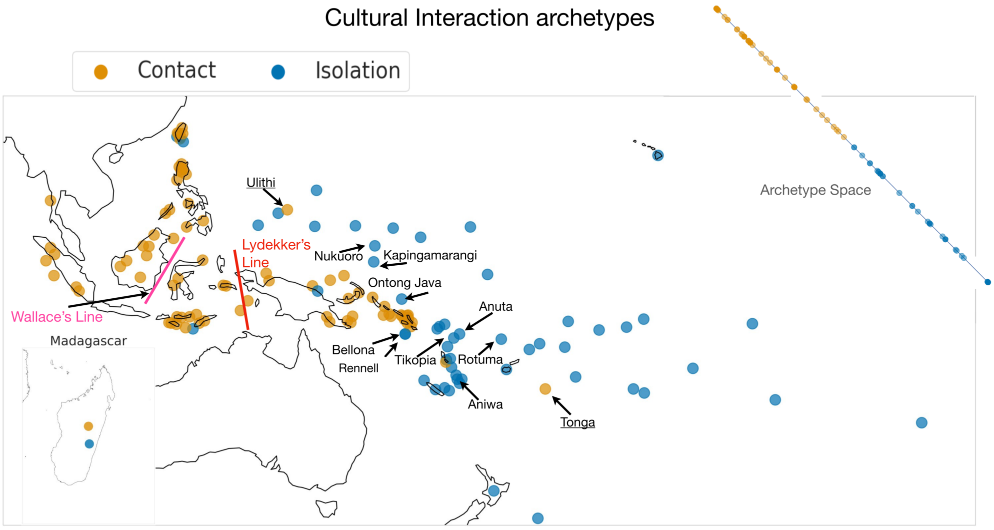

B

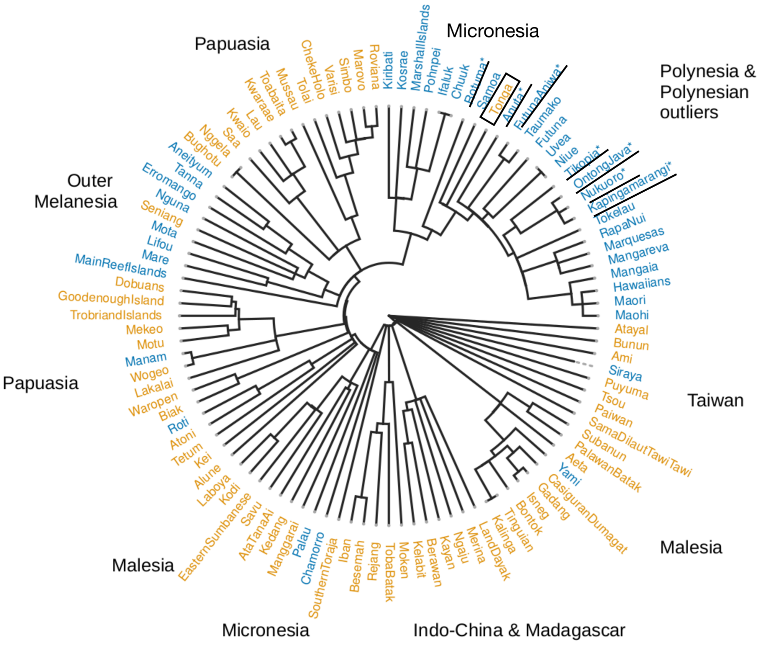

Figure S3: Variation in Cultural Interaction. Over **(a)** geography, **(inset)** archetypes (the Polynesian outliers in this data are labeled with text arrows), and **(b)** Austronesian language tree. Lydekker's line and Wallace's line are proposed geographic barriers separating the Asian and Australian biospheres (see Ali and Heaney, 2021).

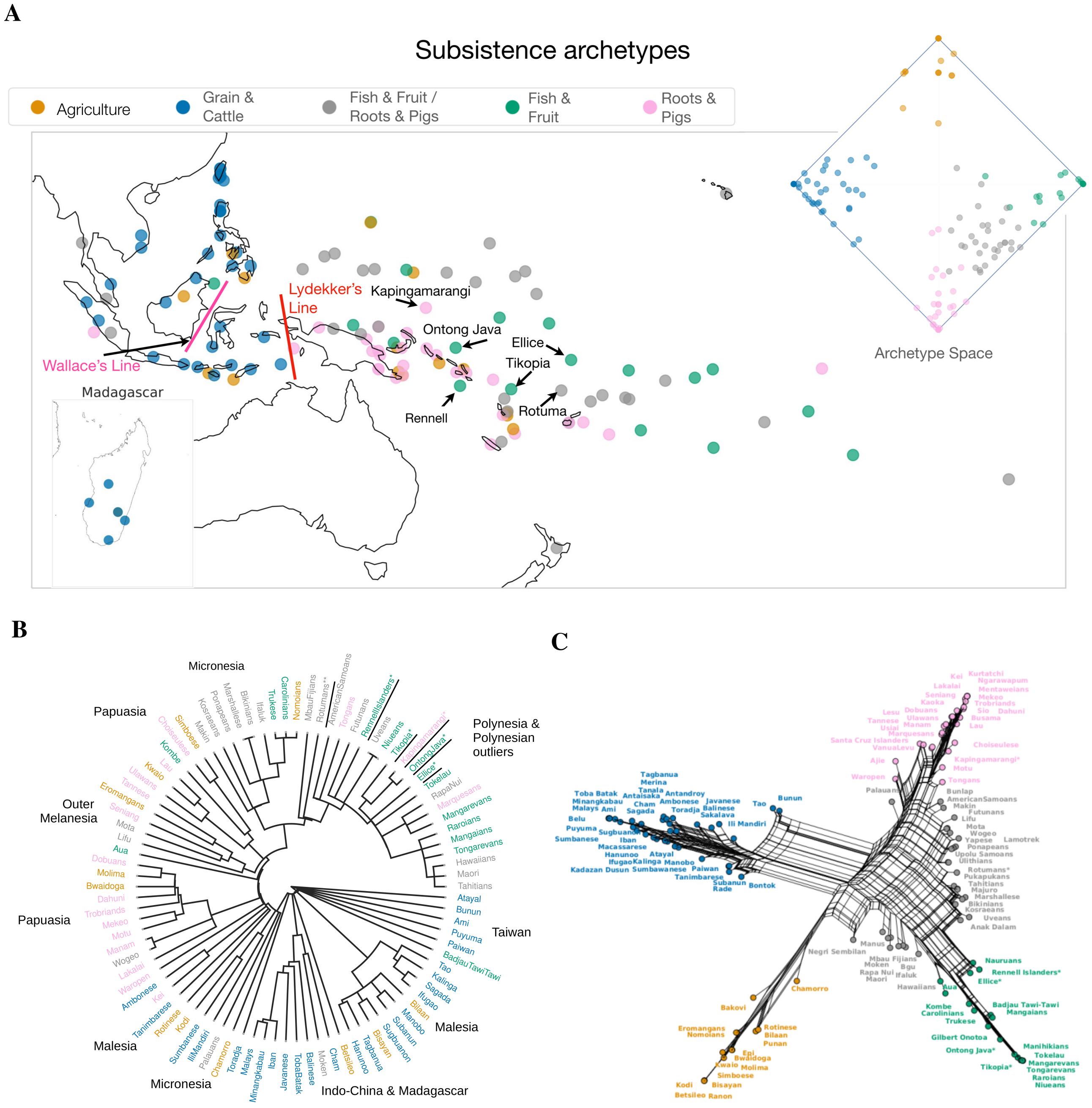

Figure S4: Variation in Subsistence. Austronesian cultures, colored by most likely cluster, over **(a)** geography, **(inset)** archetypes (the Polynesian outliers in this data are labeled with text arrows), and **(b)** Austronesian language tree. Lydekker's line and Wallace's line are proposed geographic barriers separating the Asian and Australian biospheres (see Ali and Heaney, 2021).

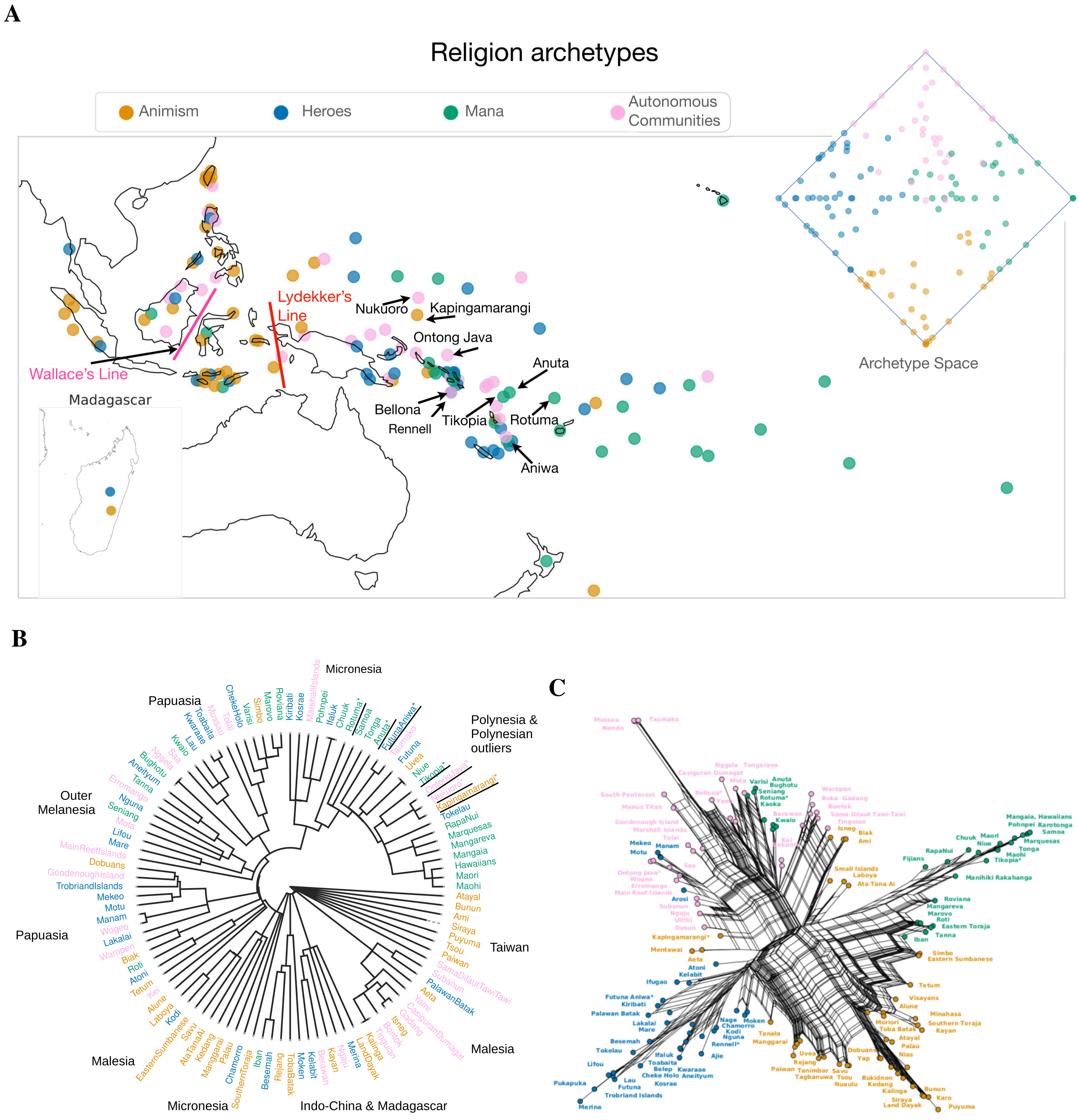

Figure S5: Variation in Religion. Austronesian cultures, colored by most likely cluster, over **(a)** geography, **(inset)** archetypes (the Polynesian outliers in this data are labeled with text arrows), and **(b)** Austronesian language tree. Lydekker's line and Wallace's line are proposed geographic barriers separating the Asian and Australian biospheres (see Ali and Heaney, 2021).

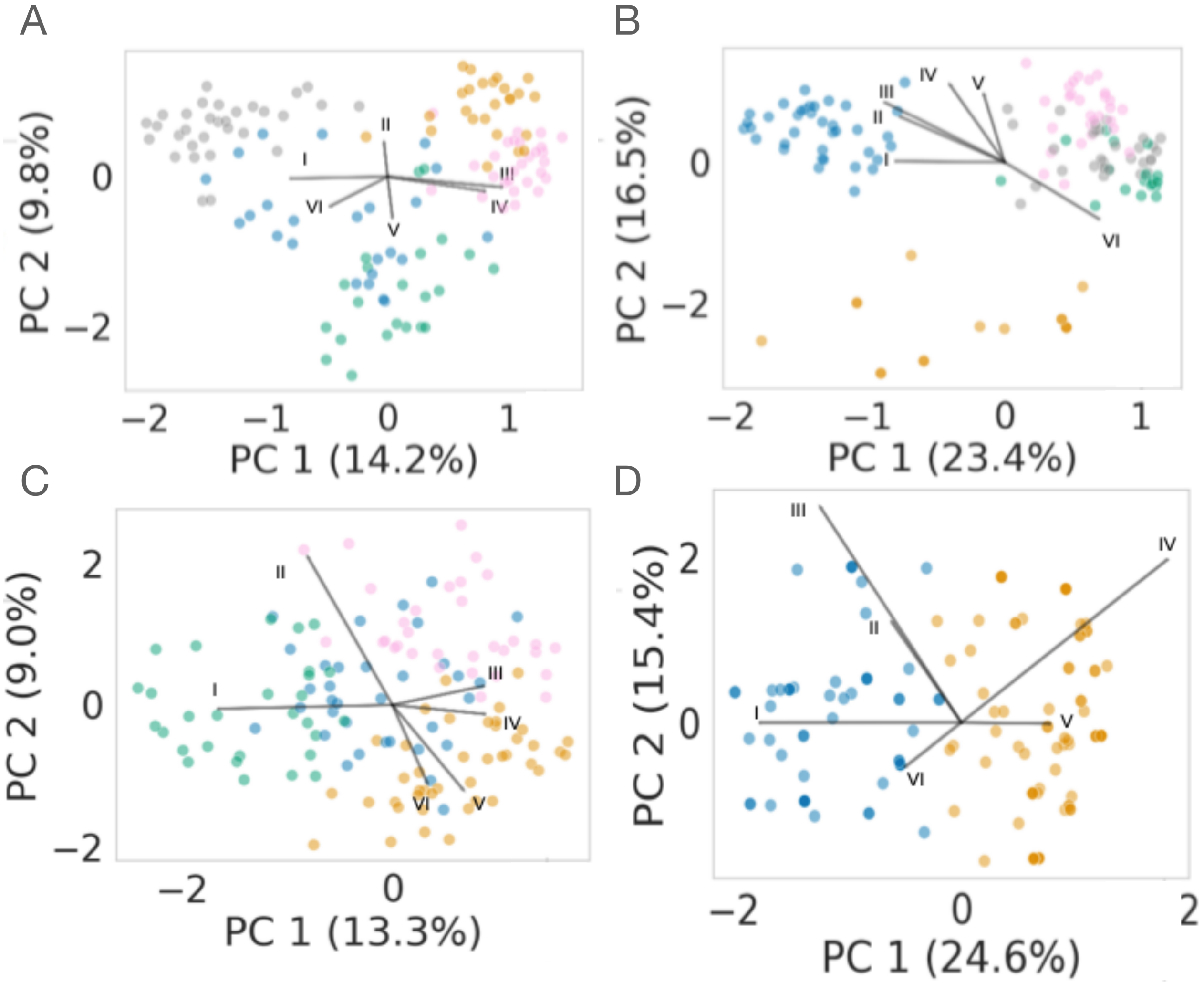

Figure S6: Two-dimensional PCA projections of the cultural data. **(A)** *Social Organization* data; loadings are: I) Token bride price, II) Polygamy, III) Significant bride price prevailing pattern, IV) Bride service, V) Domestic organization: small extended families, VI) Significant bride price, alternative pattern. Thus, PC 1 captures differences in domestic organization and PC 2 differences in transactions/exchange at marriage. **(B)** *Subsistence* data; loadings are I) Animal husbandry 46-55% of subsistence, II) Fishing 16-25% of subsistence, III) Fishing 6-15% of subsistence, IV) Agriculture 0-5% of subsistence, V) Complete absence of agriculture, VI) Fishing 36-45% of subsistence. Thus, PC 1 captures differences in the importance of animal husbandry and fishing and PC 2 differences in the importance of agriculture. **(C)** *Religion* data; loadings are I)Actions of others after one has died do not affect the nature of one's afterlife, II) No myth of man's creation, III) Belief in cultural heroes is a major focus, IV) Belief in nature spirit(s) a major focus, V) Creationist myth of humans, VI) Actions while living are the principal determinant of the nature of one's afterlife. Thus, PC 1 captures differences in religious focus, and PC 2 differences in creation myths. **(D)** *Cultural Interaction* data; loadings marked by I) Pre-Austronesians absent, II) Common conflict between communities, III) Occasional conflict between communities, IV) Frequent conflict with other societies, V) Pre-Austronesians present, VI) Common conflict with other societies. Thus, PC 1 captures the presence/absence of pre-existing populations at the time of settlement, and PC 2 captures differences in the nature and frequency of conflict within and between societies.

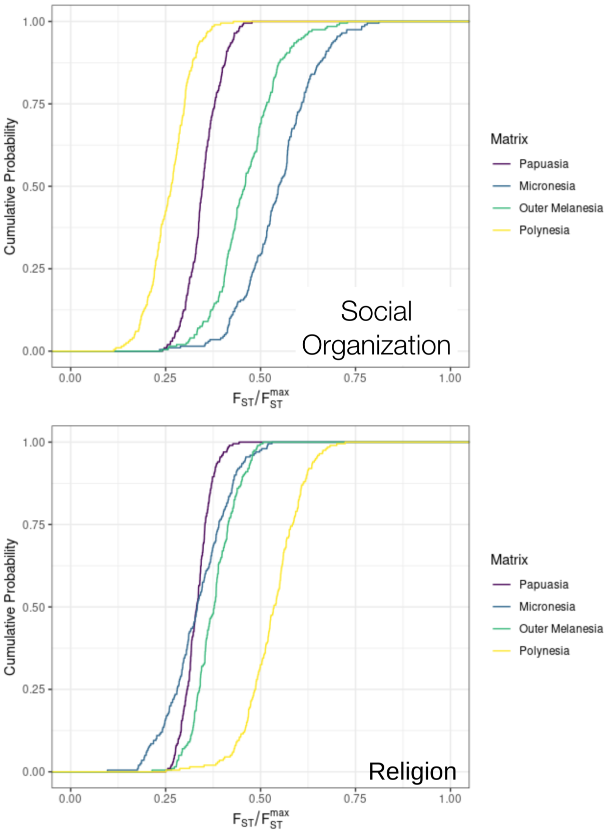

Figure S7: Within geographic region variability of archetype sets. Cumulative density plots for F_{st}/F_{st}^{max}, which is proportional to Dirichlet distribution variance in Polynesia and regions containing Polynesian outliers.

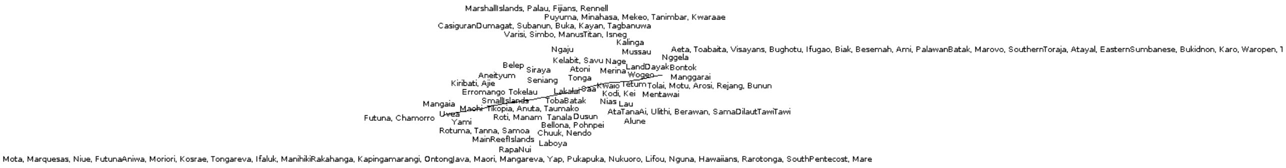

Figure S8: Cultural Interaction network. Due to the binary nature of these archetypes, the network reduces to a tree with only two branches.

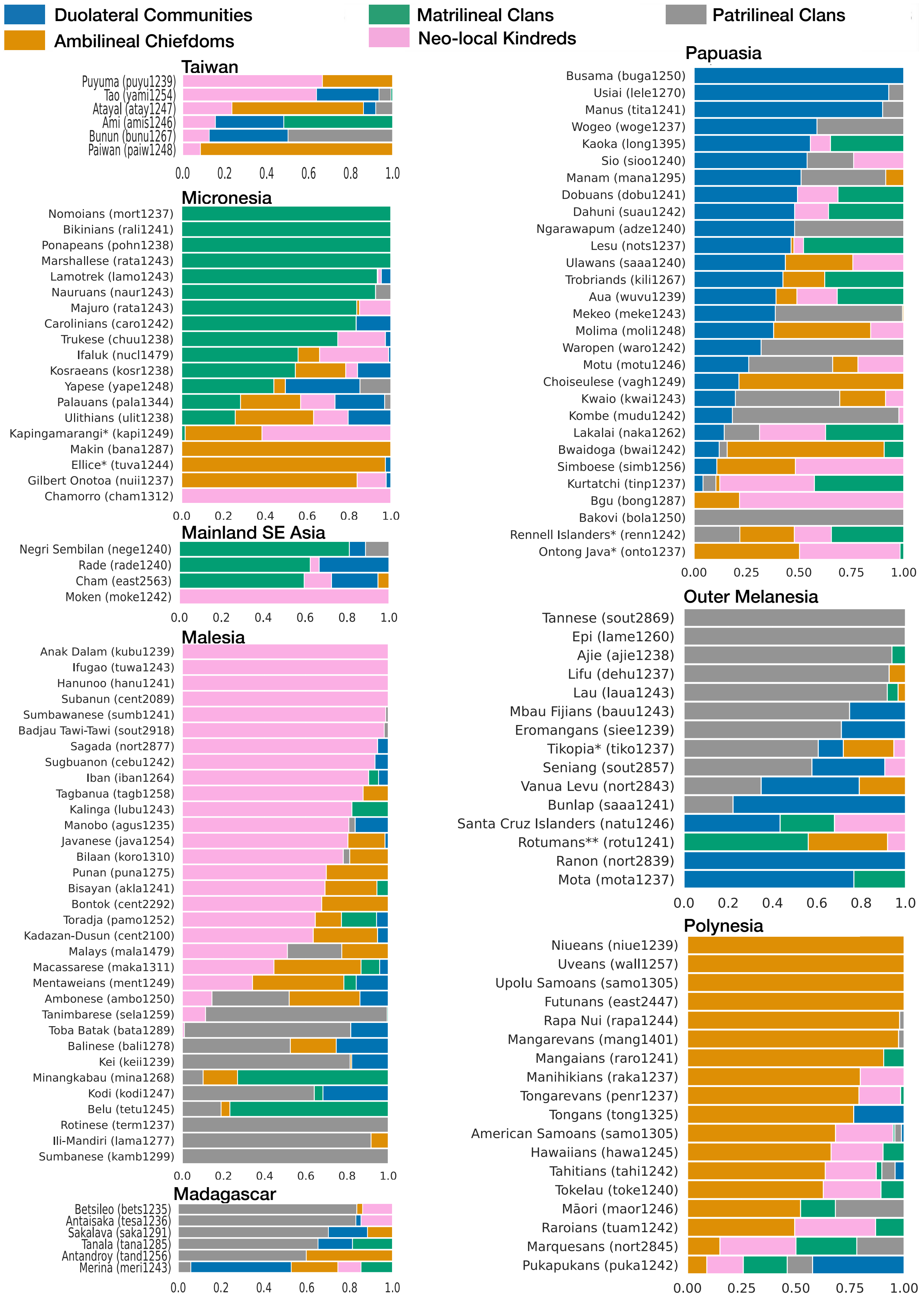

Figure S9: Social Organization bar-plots with respect to archetypes. Cultures are separated by geographic region.

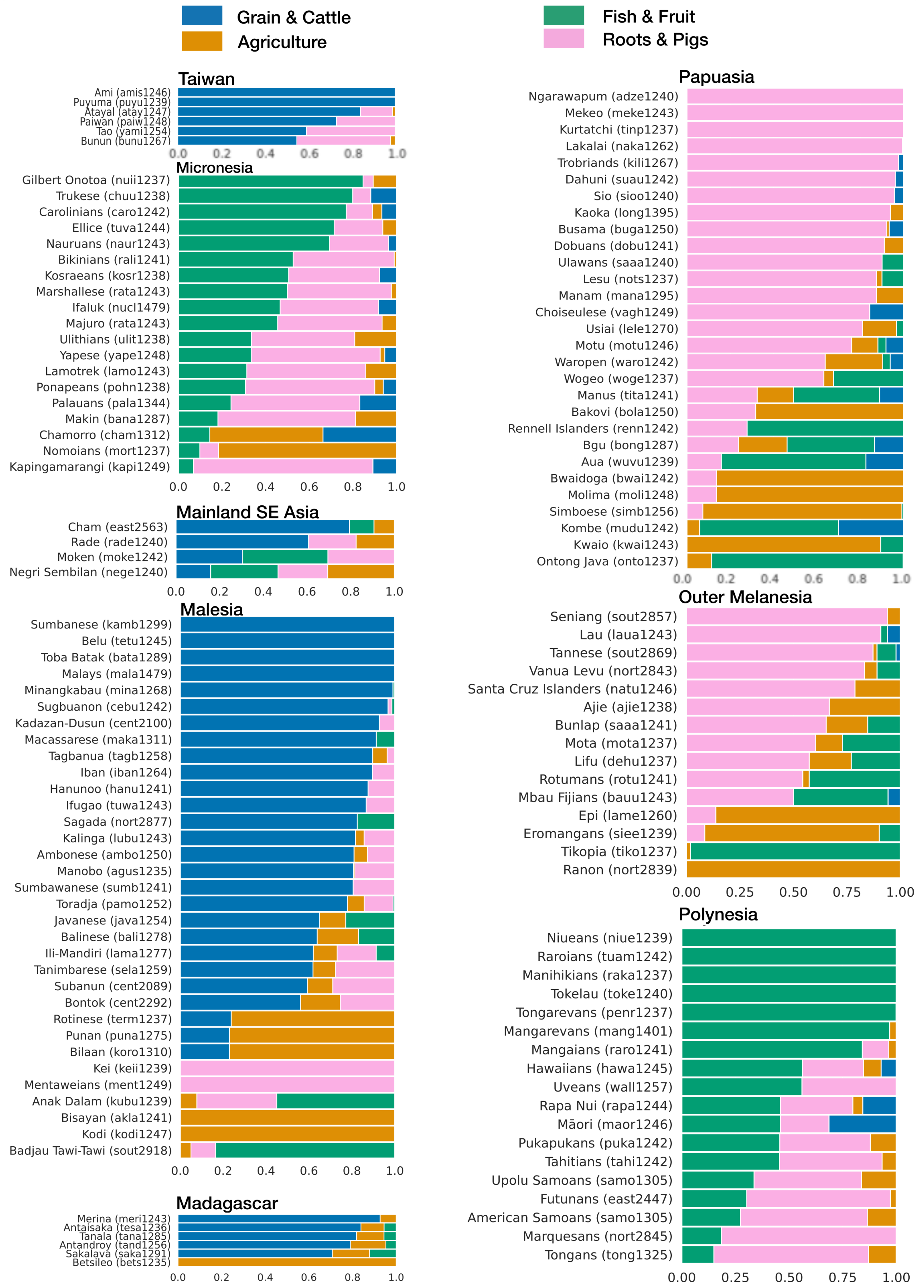

Figure S10: Subsistence bar-plots with respect to archetypes. Cultures are separated by geographic region.

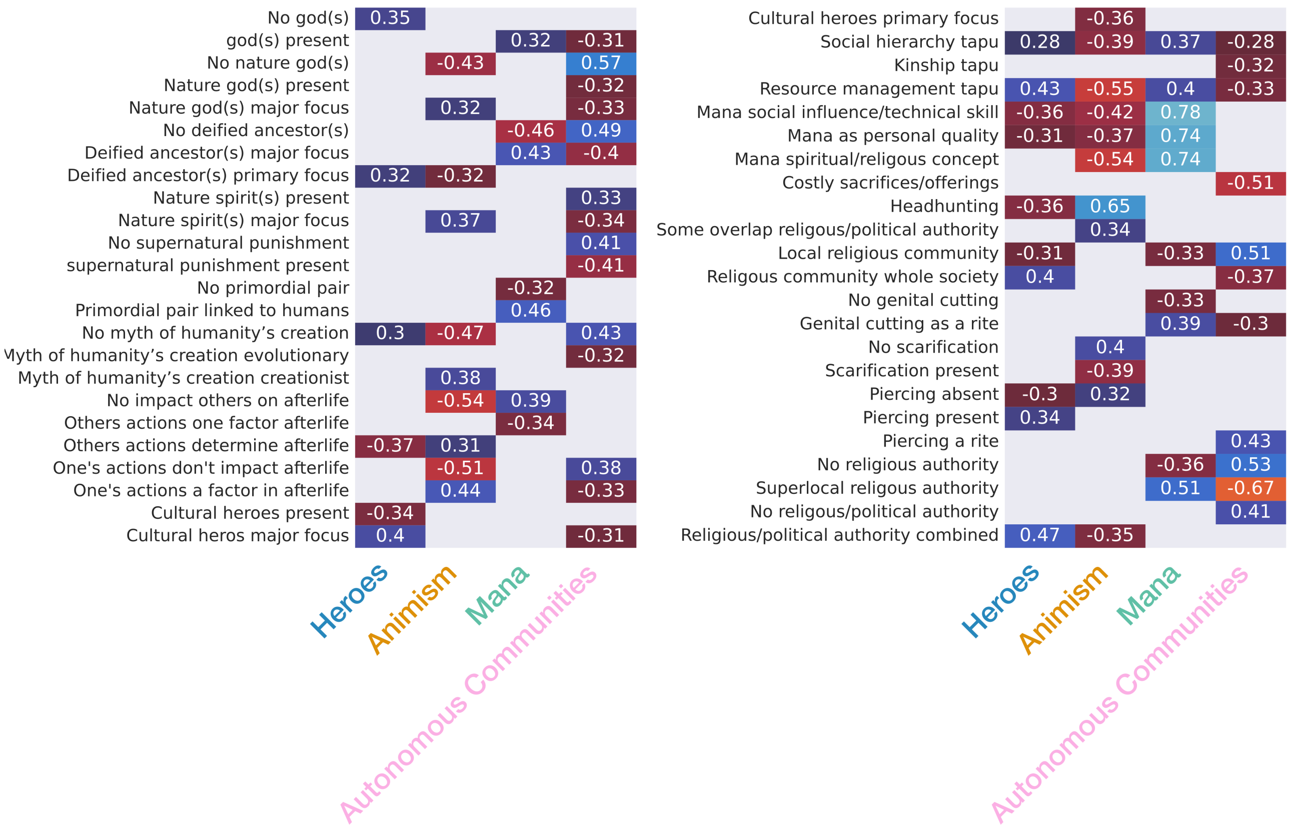

Figure S11: Binarized Religion trait correlations with archetypes. Only features (n = 89) that have correlation P-values less than 0.001 after FDR correction are displayed (n = 47).

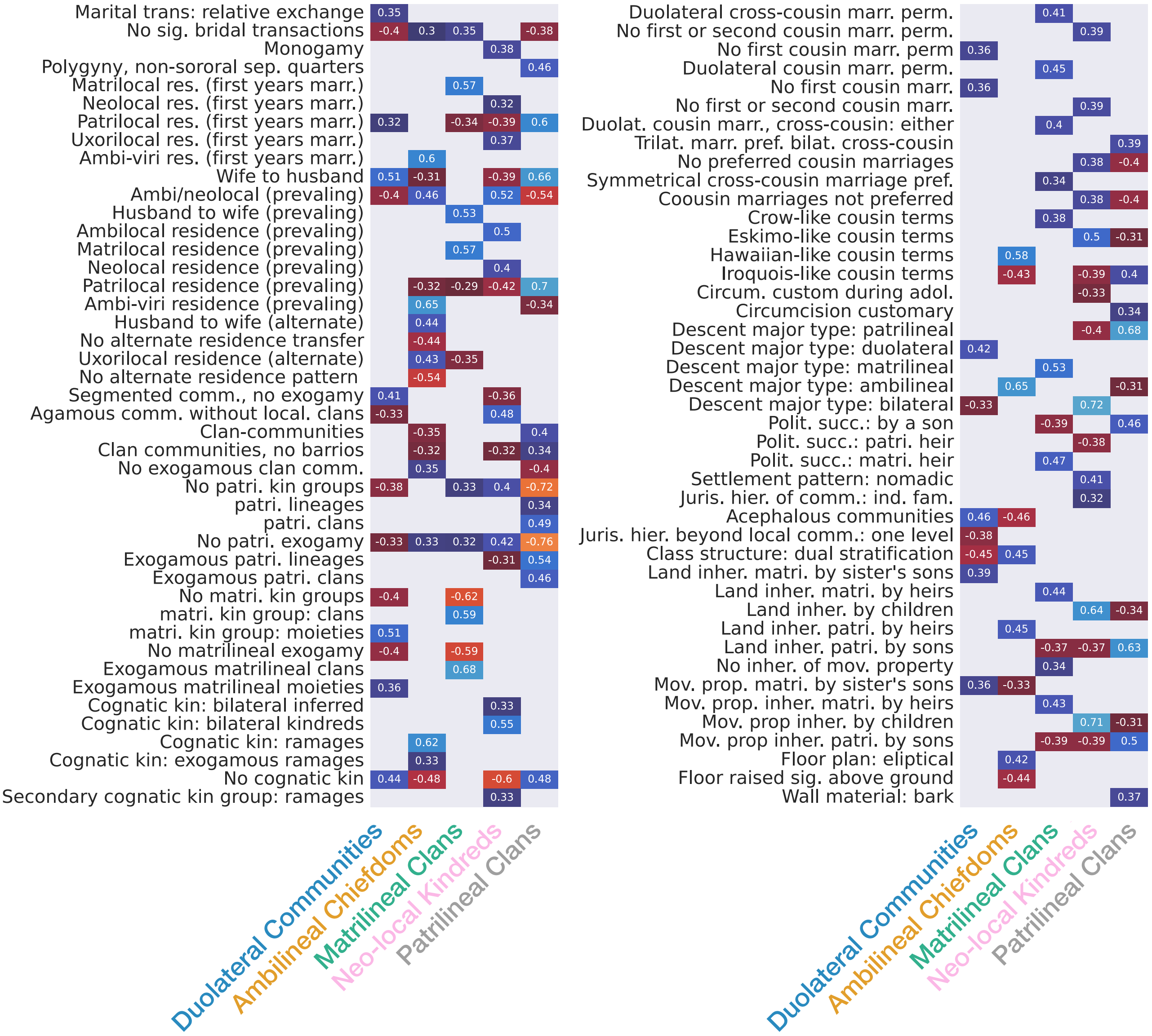

Figure S12: Binarized Social Organization trait correlations with archetypes. Only features (n = 236) that have correlation P-values less than 0.001 after FDR correction are displayed (n = 87).

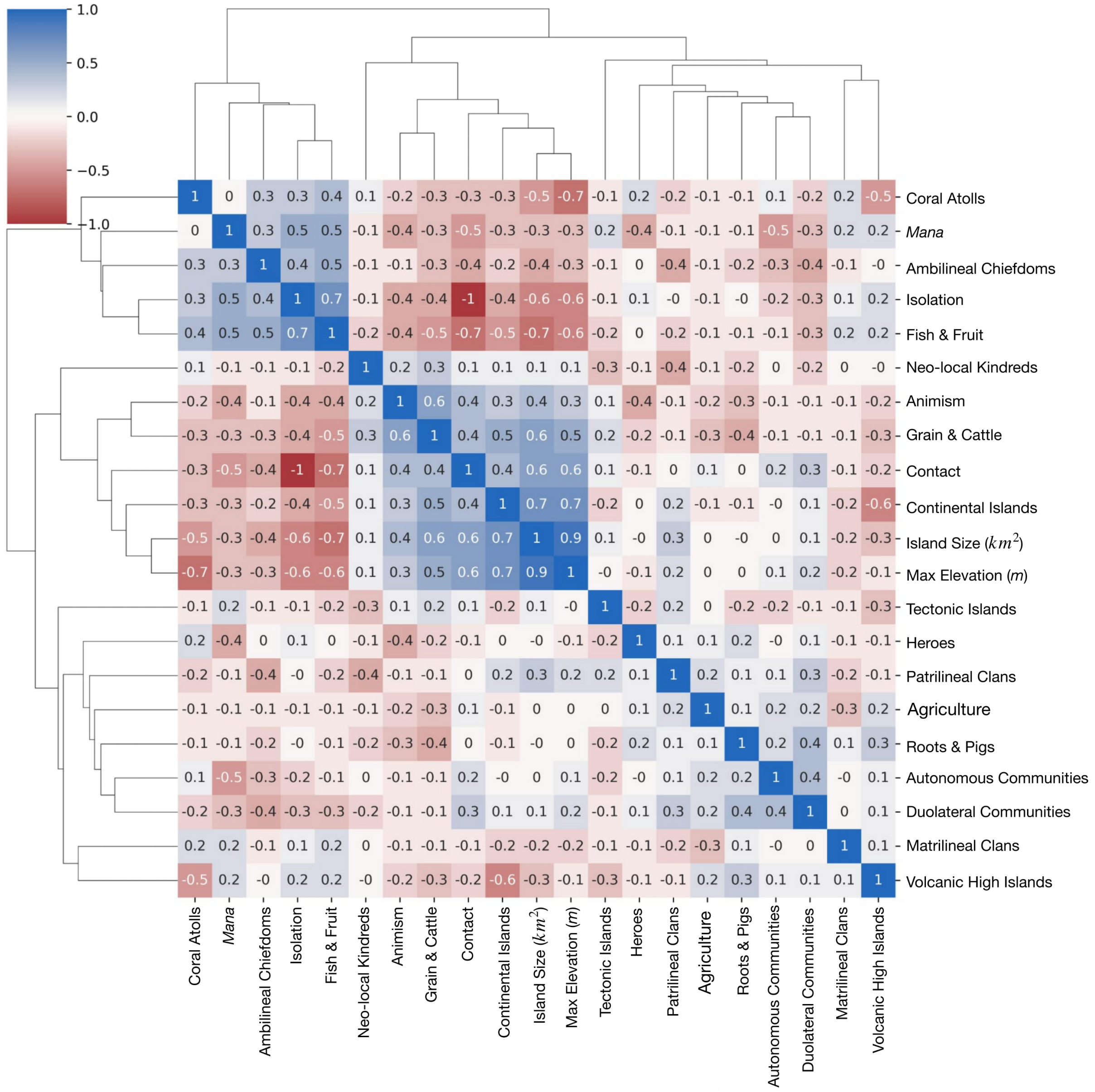

Figure S13: Hierarchical cluster-map on the Spearman correlation matrix for both archetypes and non-cultural features across the 65 cultures in common between EA and Pulotu. There are three clusters that appear to correspond to the results of Karin and Alon (2018).

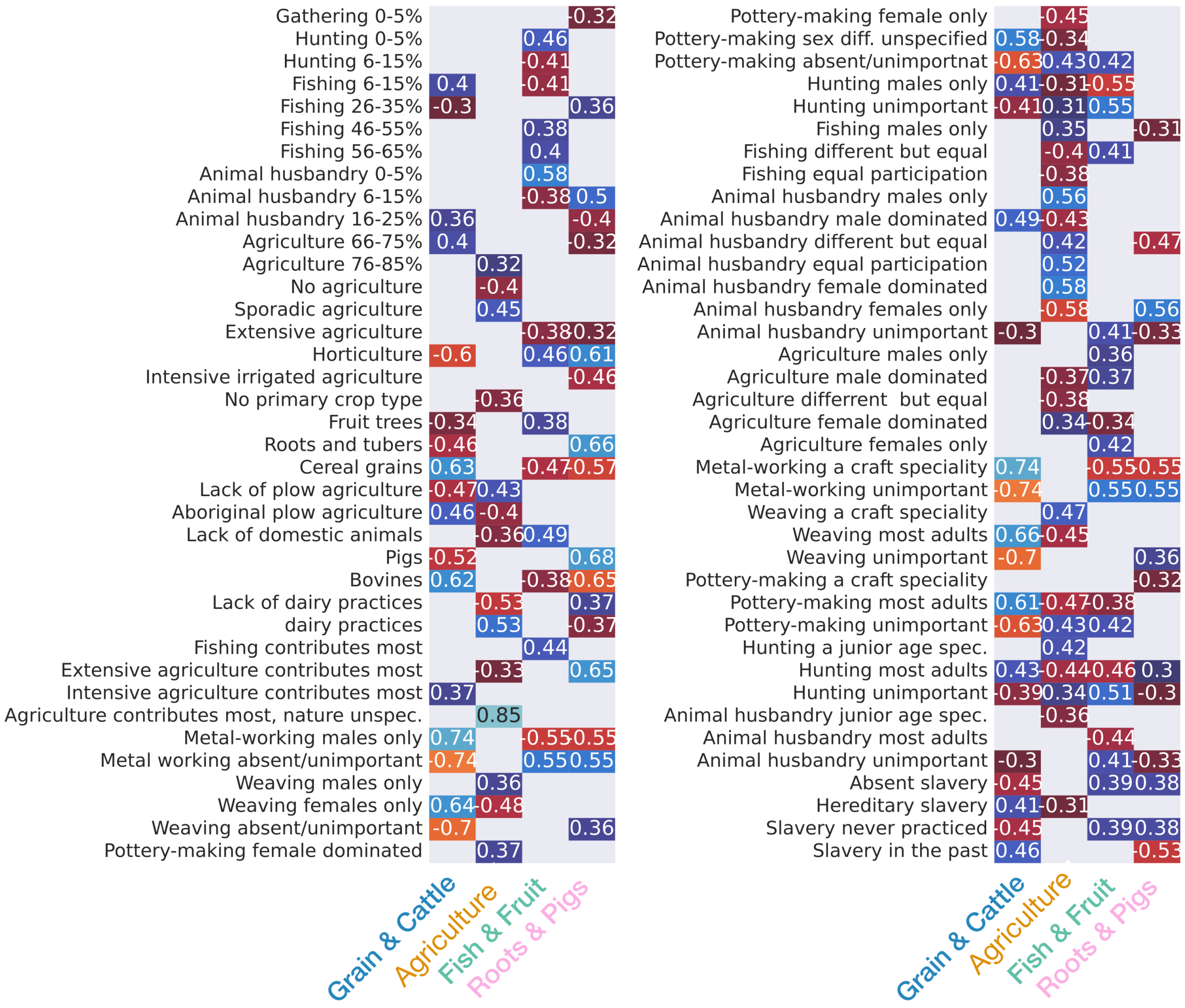

Figure S14: Binarized Subsistence trait correlations with archetypes.. Only features (n = 121) that have correlation P-values less than 0.001 after FDR correction are displayed (n = 76). Note that the Agriculture archetype is missing many features on average (Table S11) and is primarily associated with (1) this missigness and (2) agriculture of unspecified type providing most of subsistence.

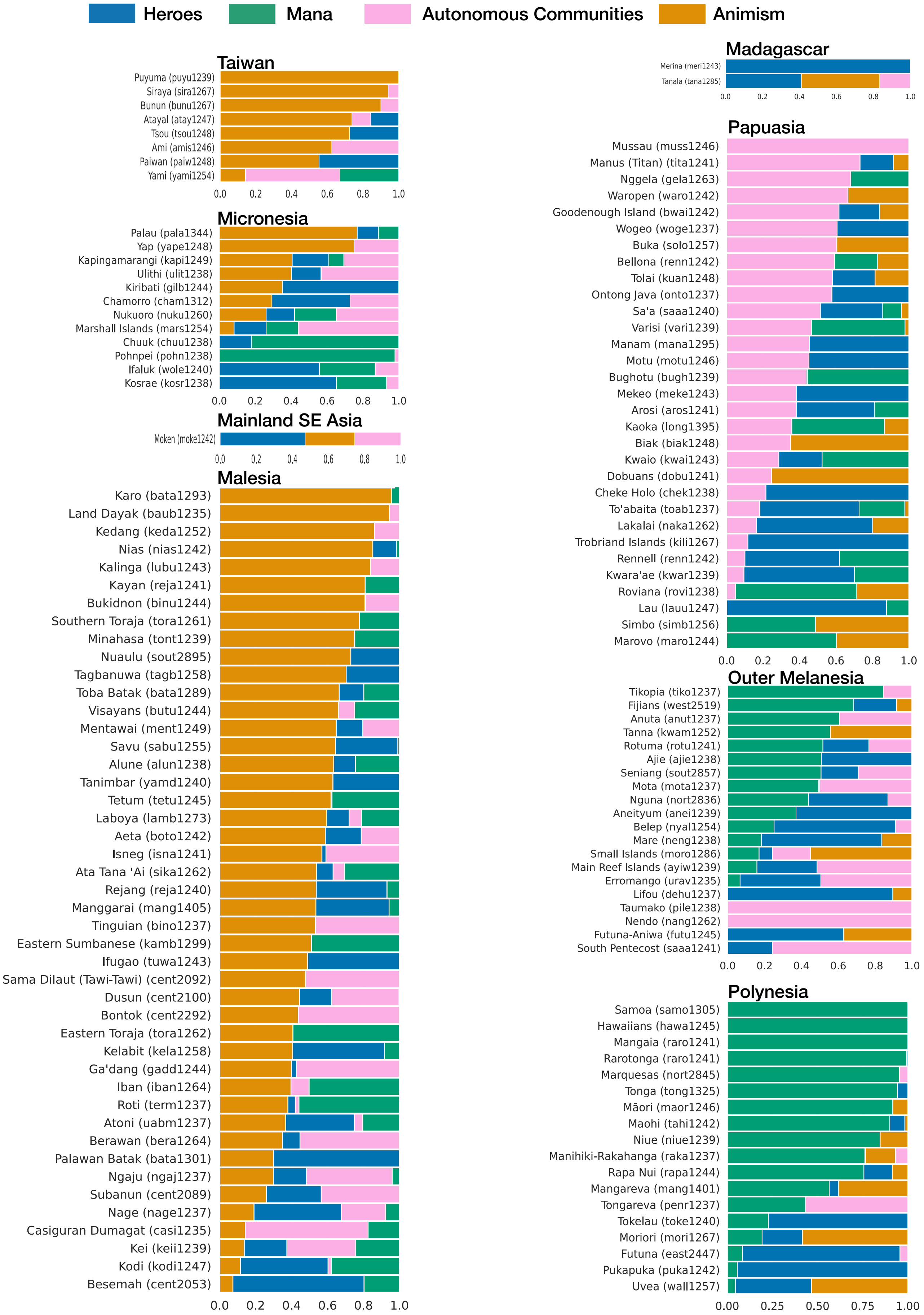

Figure S15: Religion bar-plots with respect to archetypes. Cultures are separated by geographic region.

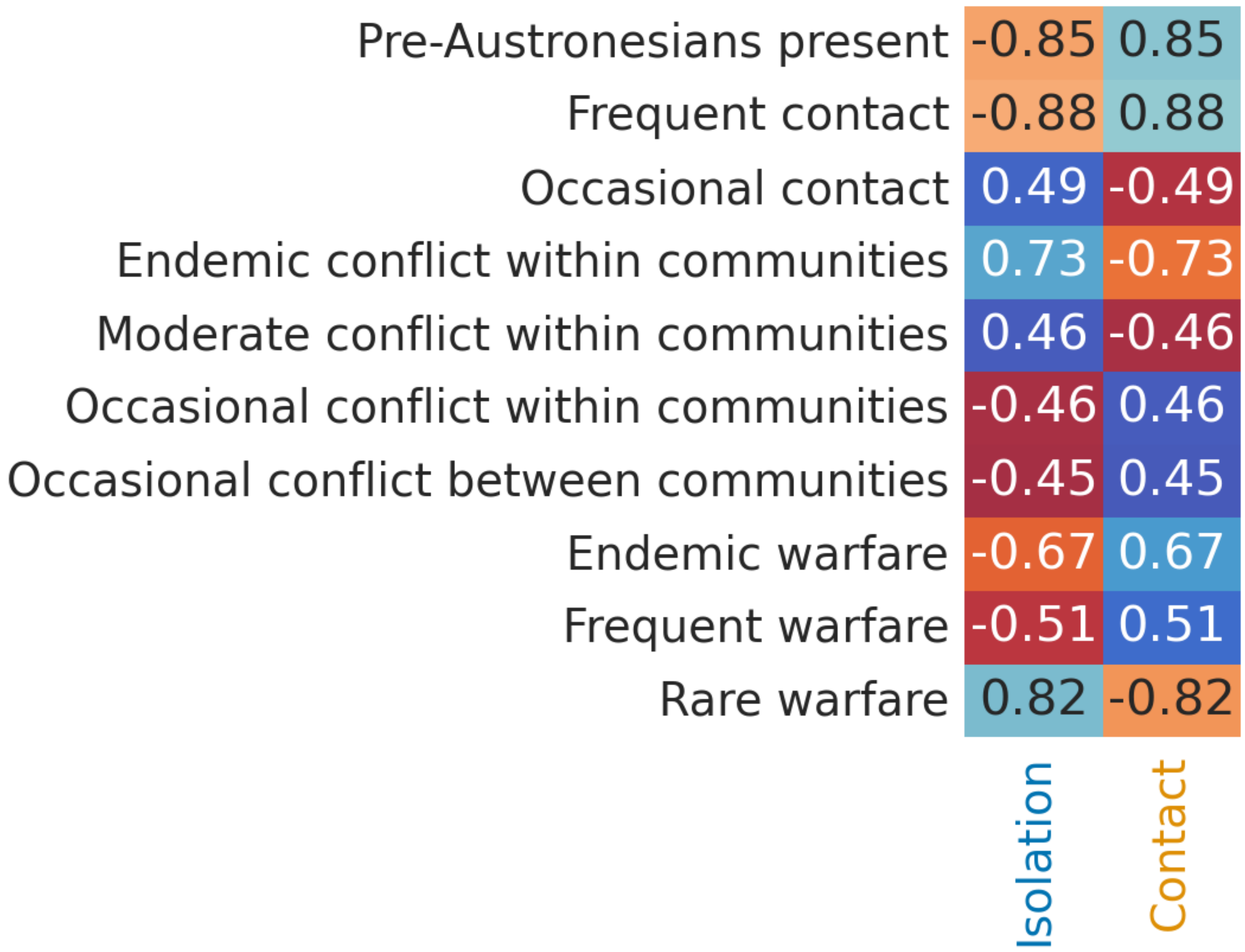

Figure S16: Binarized Cultural Interaction trait correlations with archetypes. Only features (n = 18) that have correlation P-values less than 0.001 after FDR correction are displayed (n = 10).

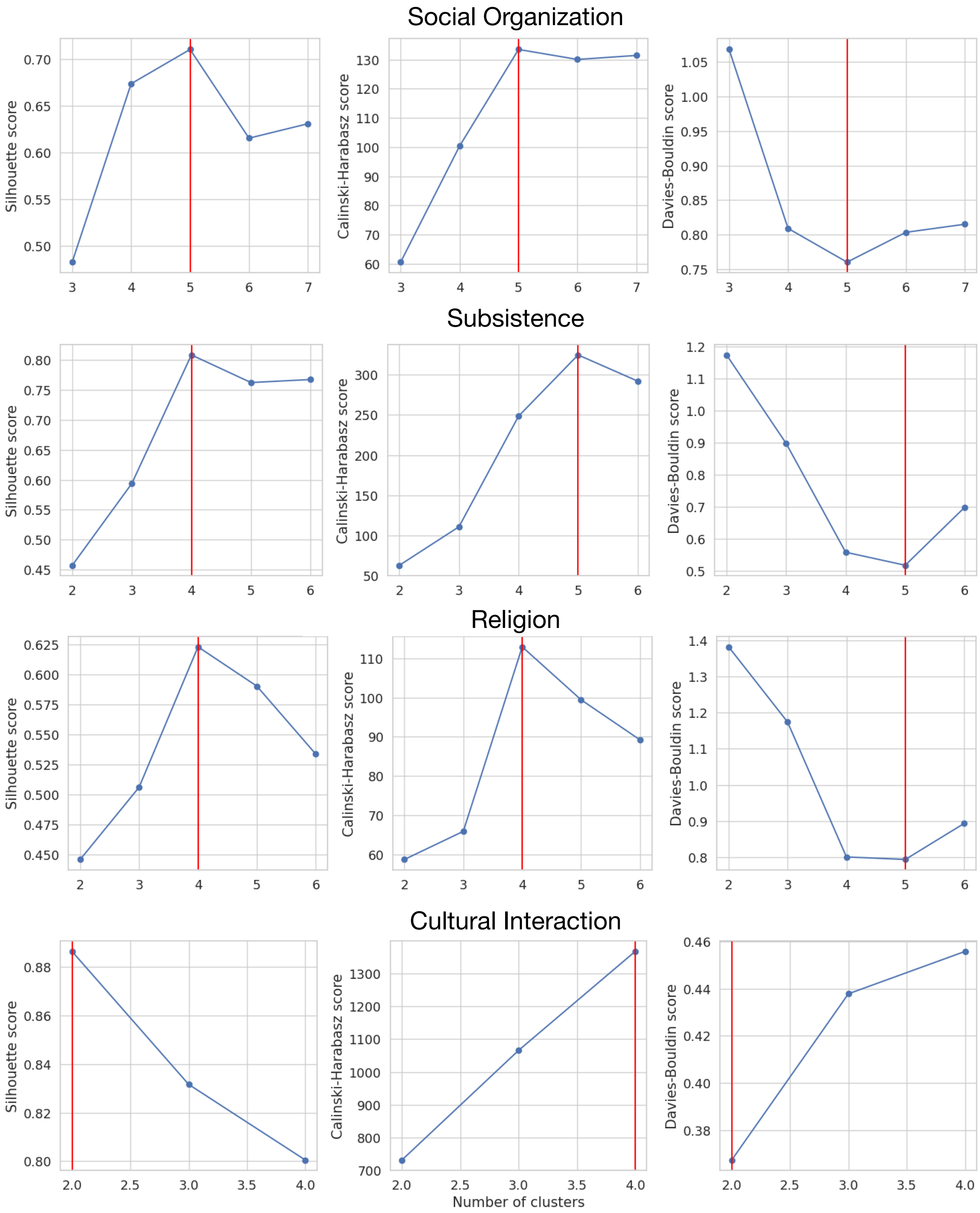

Figure S17: Kmeans clustering metrics for distances calculated in archetype space. Optimal numbers of Kmeans clusters for each data class (rows) according to the silhouette score (column 1), the variance ratio criterion (column 2), and the Davies-Bouldin score (column 3). For all archetypes except *Subsistence*, the optimal number of clusters on the archetypes is the same as the number of archetypes.

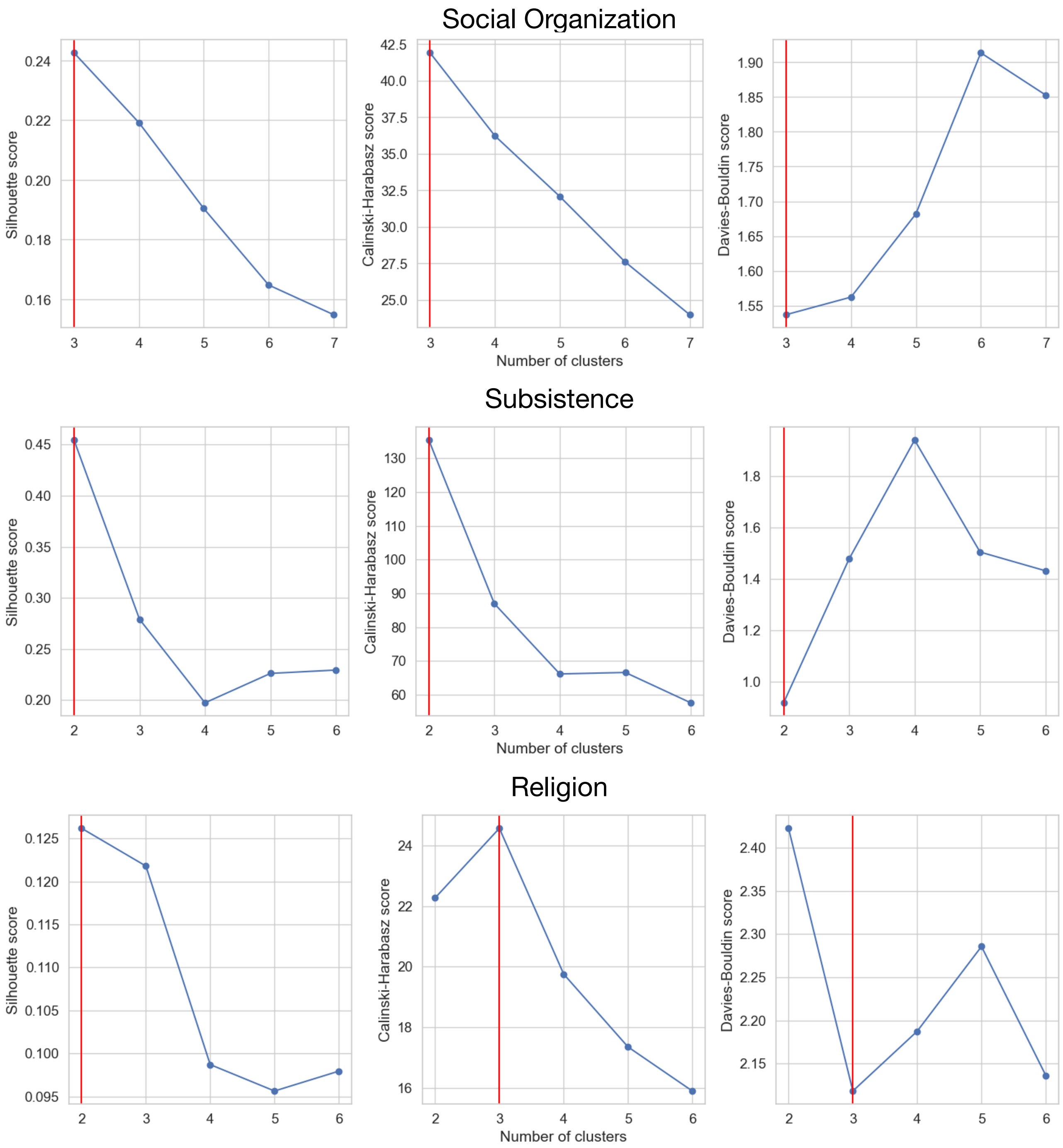

Figure S18: Kmeans clustering metrics for Hamming distances. Optimal numbers of Kmeans clusters for each data class (rows) according to the silhouette score (column 1), the variance ratio criterion (column 2), and the Davies-Bouldin score (column 3). The Hamming distance underestimates the number of clusters.

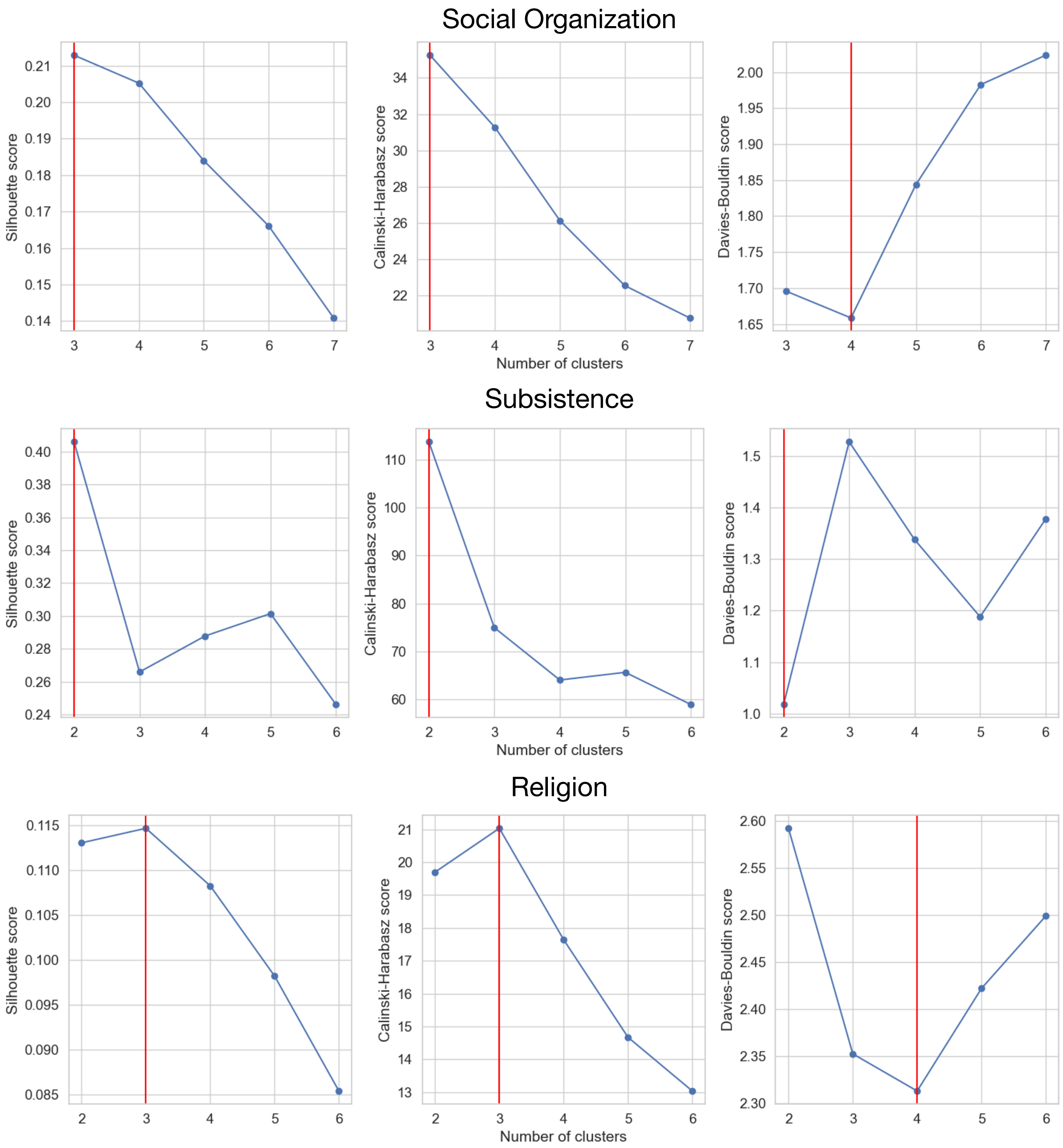

Figure S19: Kmeans clustering metrics for Jaccard distances. Optimal numbers of Kmeans clusters for each data class (rows) according to the silhouette score (column 1), the variance ratio criterion (column 2), and the Davies-Bouldin score (column 3). The Jaccard distance underestimates the number of clusters.

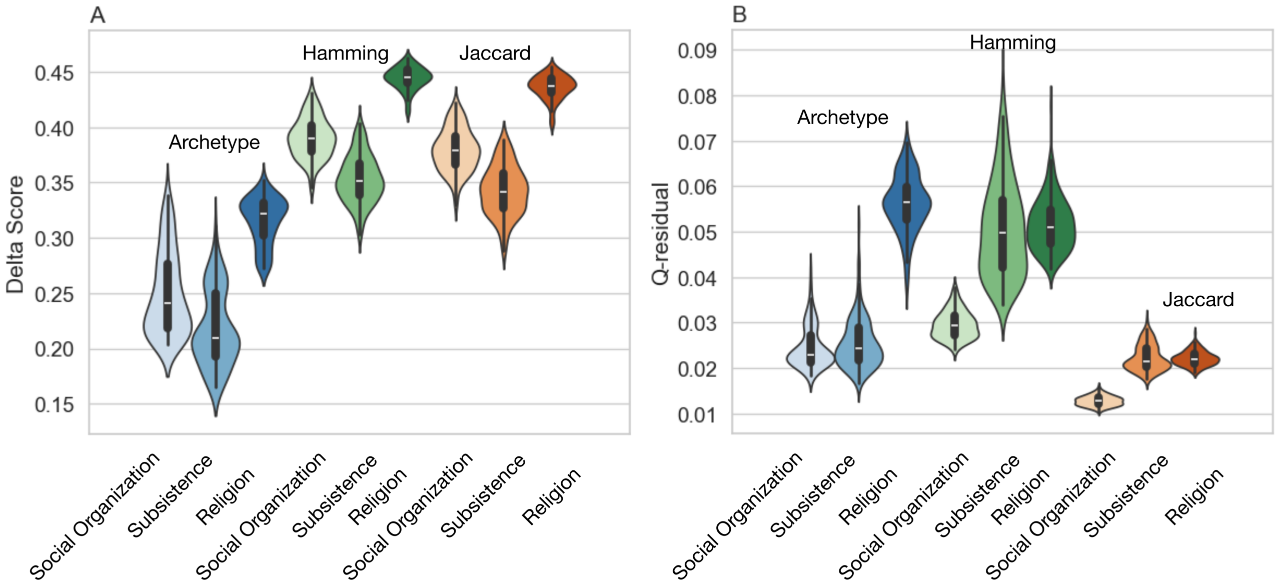

Figure S20: Comparison of δ-score and Q-residuals. The δ-score preserves relative differences in tree-likeness across choice of pairwise distance, but the Q-residual does not.

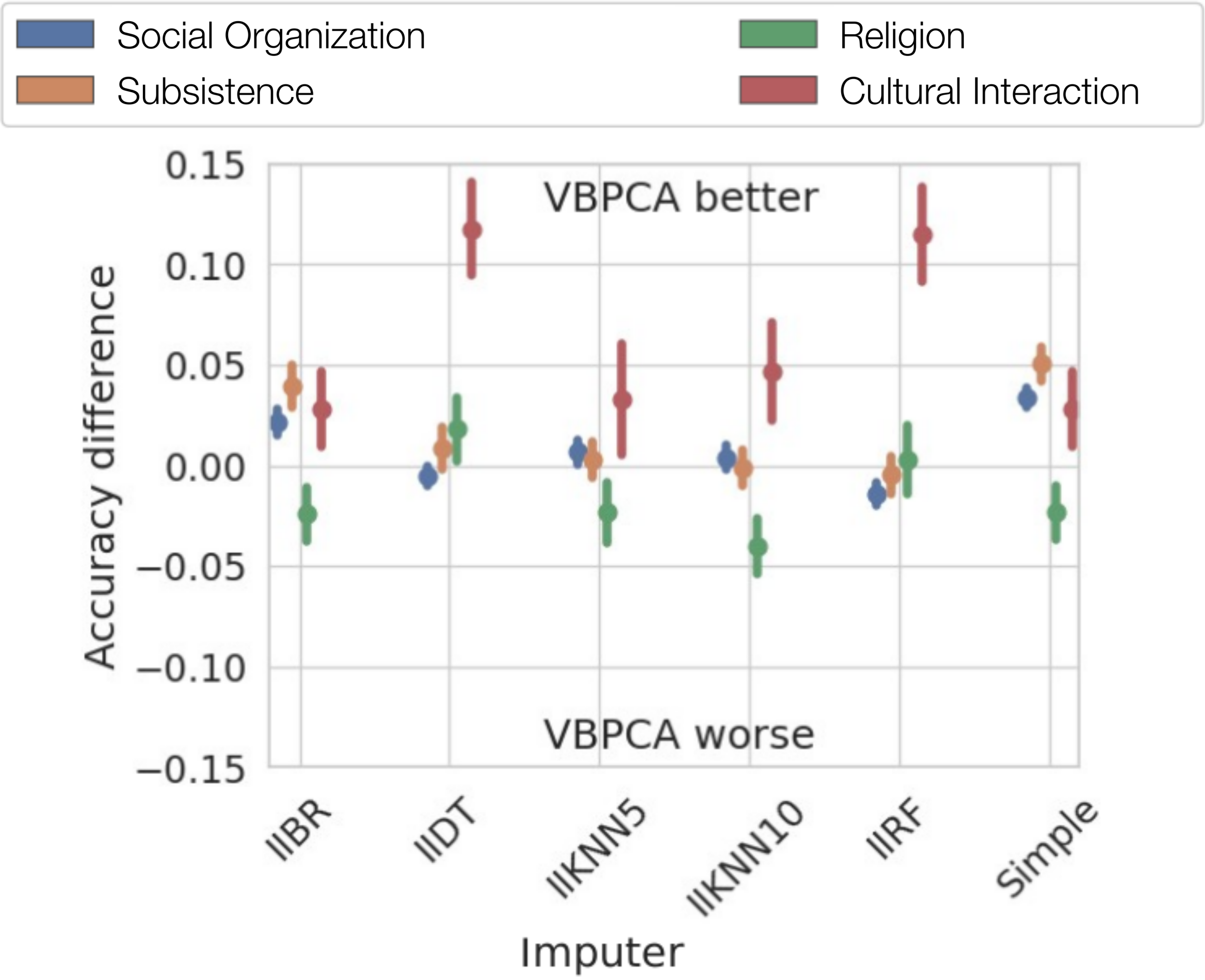

Figure S21: Imputation performance comparison. Accuracy of VBPCA imputation relative to other imputation algorithms across 50 replicates, each with 2% values deleted randomly from one of the four cultural class datasets. Given that none of the imputers consistently outperforms VBPCA and that VBPCA provides marginal posterior estimates for the data matrix (which other imputers do not), VBPCA is used for the remainder of our analysis. Positive values indicate that VBPCA performs better. The vertical lines are 95% confidence intervals for the difference in accuracy. The alternative imputers are Iterative imputer with one of the following regressors: Bayesian ridge regressor (IIBR), Iterative imputer with decision tree regressor (IIDT), Iterative imputer with nearest neighbor regressor with $k = 5$ (IIKNN5) and $k = 10$ (IIKNN10), Iterative imputer random forest regressor (IIRF), and simple imputation with missing data replaced by column means (Simple).

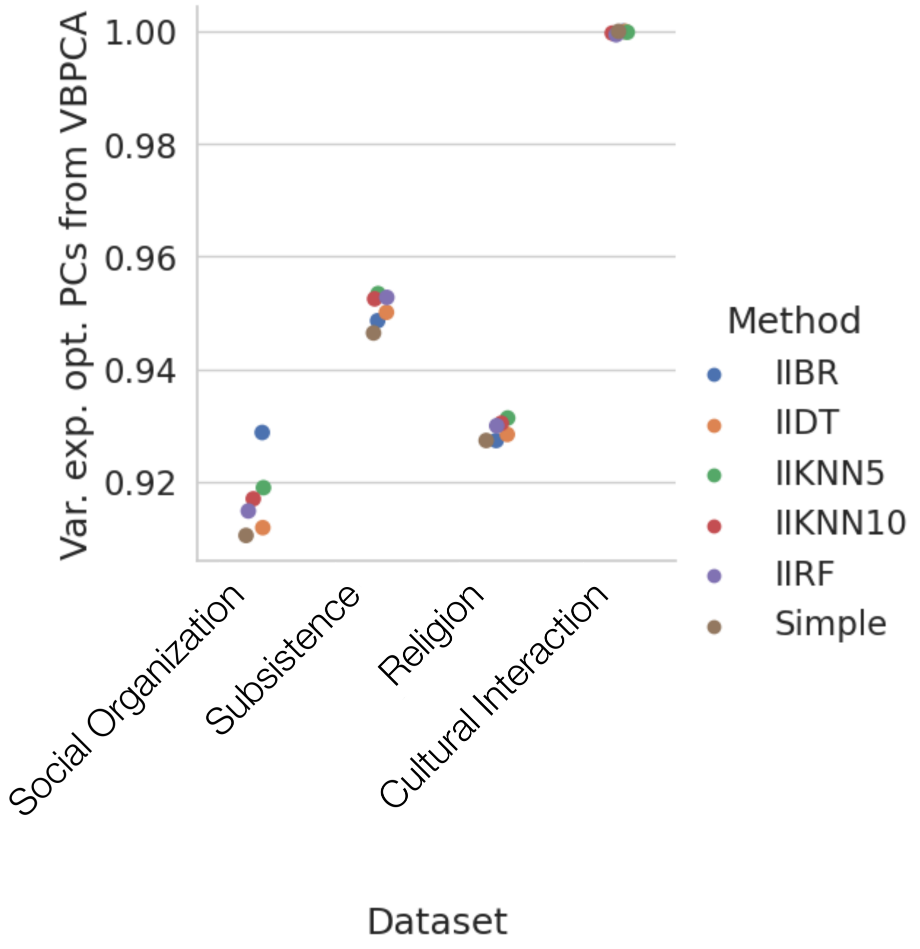

Figure S22: VBPCA noise filtering for different datasets. The proportion of cumulative variance explained by the optimal number of principal components for data reconstruction as determined by VBPCA using classical PCA after filling missing values via different imputation approaches.

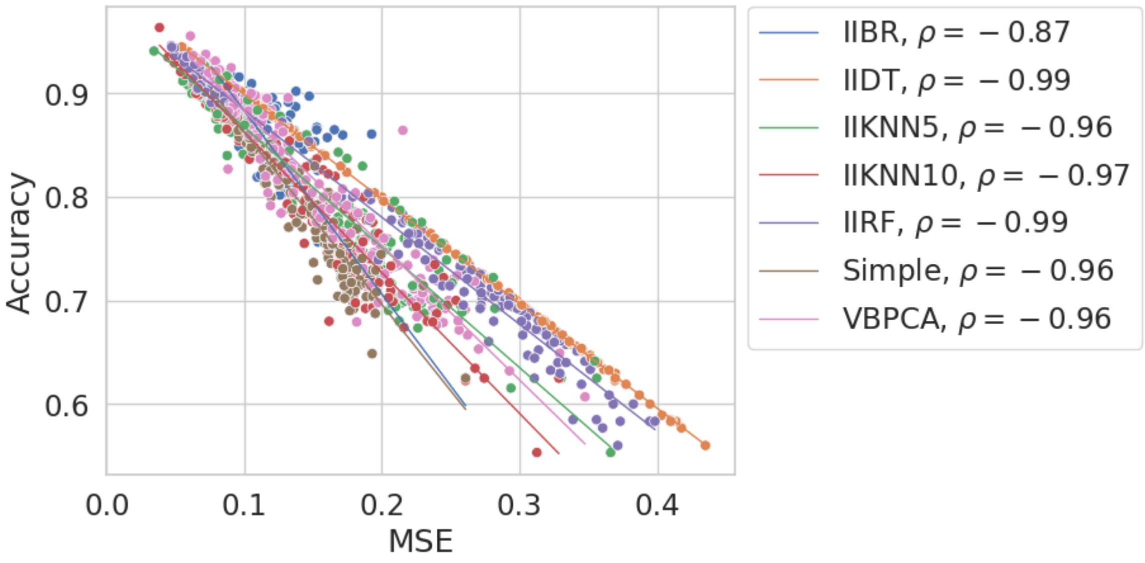

Figure S23: The relationship between Accuracy and MSE. Across all datasets and imputers, there is a strong correlation between MSE (mean squared error) and the accuracy of the inferred values.

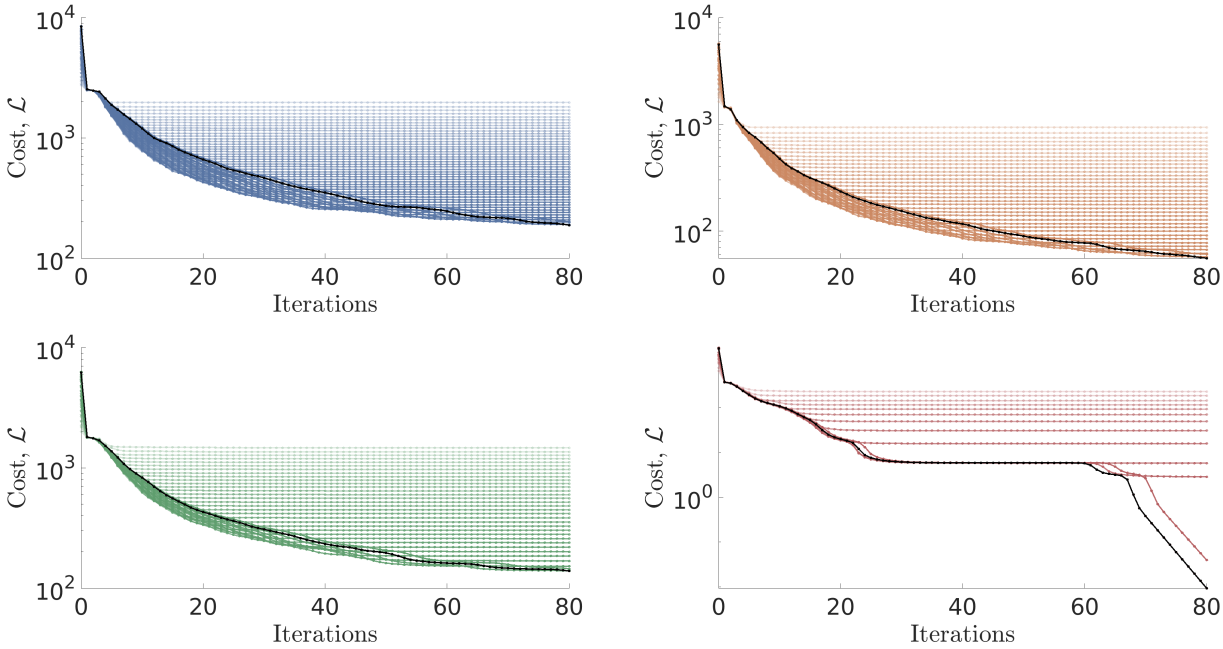

Figure S24: Cost functions for each of the four cultural classes. The colors of the lines get darker as the number of principal components increases, and the optimal number of PCs (49, 37, 34, 13 for each of the datasets) is colored in black. As the optimum is approached, the values of the cost function for a given number of principal components begin to 'stack up.'

Table S1: Distance to nearest neighbor (km) by geographic region. A value of 0 indicates immediate adjacency to the nearest neighbor.

Region	Sample mean	Standard error
Malesia	5.71	2.71
Polynesia	747.70	220.74
Papuasia	9.88	3.41
Outer Melanesia	56.50	15.69
Taiwan	7.75	7.75
Micronesia	236.51	46.12
Madagascar	0.00	0.00
Mainland SE Asia	0.00	–

Table S2: Likelihood ratio test for regional archetype distributions for geographic regions containing Polynesian outliers and Polynesia.

Archetype set	Region	LR statistic	p-value
Social Organization	Papuasia	51.0	9×10^{-10}
	Micronesia	36.4	8×10^{-07}
	Outer Melanesia	45.1	1×10^{-8}
Subsistence	Papuasia	32.3	2×10^{-6}
	Micronesia	11.9	0.0183
	Outer Melanesia	22.9	0.00013
Religion	Papuasia	38.5	9×10^{-8}
	Micronesia	17.1	0.0018
	Outer Melanesia	14.7	0.0055
Cultural Interaction	Papuasia	39.4	2.7×10^{-9}
	Micronesia	1.3	0.53
	Outer Melanesia	2.9	0.23

Table S3: Pairwise comparisons of F_{st}/F_{st}^{max} for *Social Organization* archetypes using Wilcoxon rank sum test with continuity correction.

	Papuasia	Micronesia	Outer Melanesia
Micronesia	< 2e-16	-	-
Outer Melanesia	< 2e-16	< 2e-16	-
Polynesia	< 2e-16	< 2e-16	< 2e-16

Table S4: Pairwise comparisons of F_{st}/F_{st}^{max} for *Subsistence* archetypes using Wilcoxon rank sum test with continuity correction.

	Papuasia	Micronesia	Outer Melanesia
Micronesia	< 2e-16	-	-
Outer Melanesia	< 2e-16	< 2e-16	-
Polynesia	< 2e-16	< 2e-16	< 2e-16

Table S5: Pairwise comparisons of F_{st}/F_{st}^{max} for *Religion* archetypes using Wilcoxon rank sum test with continuity correction.

	Papuasia	Micronesia	Outer Melanesia
Micronesia	0.7	-	-
Outer Melanesia	< 2e-16	< 1.4e-08	-
Polynesia	< 2e-16	< 2e-16	< 2e-16

Table S6: Pairwise comparisons of F_{st}/F_{st}^{max} for *Cultural Interaction* archetypes using Wilcoxon rank sum test with continuity correction.

	Papuasia	Micronesia	Outer Melanesia
Micronesia	0.0028	-	-
Outer Melanesia	< 2e-16	< 2e-16	-
Polynesia	< 2e-16	1.3e-14	3.3e-11

Table S7: Normalized F_{st} values for regions containing Polynesian outliers and Polynesia itself.

Archetype set	Region	F_{st}/F_{st}^{max}	sample size	number archetypes
Social Organization	Polynesia	0.268	18	5
	Micronesia	0.571	19	5
	Outer Melanesia	0.478	15	5
	Papuasia	0.360	29	5
Subsistence	Polynesia	0.338	18	4
	Micronesia	0.253	19	4
	Outer Melanesia	0.456	15	4
	Papuasia	0.565	29	4
Religion	Polynesia	0.560	18	4
	Micronesia	0.352	12	4
	Outer Melanesia	0.392	20	4
	Papuasia	0.339	31	4
Cultural Interaction	Polynesia	0.281	18	2
	Micronesia	0.366	12	2
	Outer Melanesia	0.194	20	2
	Papuasia	0.401	31	2

Table S8: Mann-Whitney U test, the difference in log-likelihood; alternative hypothesis Polynesian likelihood is greater. * indicates statistical significance.

Archetype set	Culture	MW statistic	p-value
Social Organization	Ellice* (Micronesia)	28508	9×10^{-14}
	Kapingamarangi* (Micronesia)	24235	0.00012
	Ontong Java* (Papuasia)	26453	1×10^{-8}
	Rennell Islanders* (Papuasia)	36452	3×10^{-46}
	Rotumans* (Outer Melanesia)	27227	2×10^{-10}
	Tikopia (Outer Melanesia)	16235	0.999
Subsistence	Ellice	18350	0.923
	Kapingamarangi	9924	1.0
	Ontong Java*	32412	3×10^{-27}
	Rennell Islanders*	27560	3×10^{-11}
	Rotumans*	21982	0.0433
	Tikopia*	26947	9×10^{-14}

Table S9: Mann-Whitney U test, the difference in log-likelihood; alternative hypothesis Polynesian likelihood is greater. * indicates statistical significance.

Archetype set	Culture	MW statistic	p-value
Religion	Anuta (Outer Melanesia)	18451	0.910
	Bellona (Papuasia)	13909	1.0
	Aniwa (Outer Melanesia)	15734	1.0
	Kapingamarangi	10189	1.0
	Nukuoro (Micronesia)	14961	1.0
	Ontong Java	12671	1.0
	Rennell	21614	0.0815
	Rotuma	18600	0.887
	Tikopia	21826	0.0572
Cultural Interaction	Bellona	713	1.0
	Ontong Java*	36648	3×10^{-47}
	Rennell	1071	1.0

Table S10: Differences in relative verticality of transmission among cultural archetype sets as indicated by Dunn's test for pairwise differences with Holm's correction for the p-values.

Metric		Social Organization	Subsistence	Religion
	Social Organization	1.0	$p < 1 \times 10^{-5}$	$p < 1 \times 10^{-5}$
δ−score	Subsistence	$p < 1 \times 10^{-5}$	1.0	$p < 1 \times 10^{-5}$
	Religion	$p < 1 \times 10^{-5}$	$p < 1 \times 10^{-5}$	1.0
	Social Organization	1.0	0.372	$p < 1 \times 10^{-5}$
Q-residual	Subsistence	0.372	1.0	$p < 1 \times 10^{-5}$
	Religion	$p < 1 \times 10^{-5}$	$p < 1 \times 10^{-5}$	1.0

Table S11: Comparison of proportion of missing data within different archetypes and for linguistic outliers. Alone among archetypes, cultures associated with the Agriculture archetype are missing large numbers of features.

Category	Median (in cat.)	Median (out of cat.)	P-value
Social Organization			
Outlier	0.083	0.071	0.68
Ambilineal Chiefdoms	0.095	0.060	0.48
Duolateral Communities	0.071	0.095	0.26
Matrilineal Clans	0.083	0.071	0.85
Neo-local Kindreds	0.024	0.083	0.37
Patrilineal Clans	0.048	0.071	0.19
Subsistence			
Outlier	0.074	0.074	0.87
Agriculture	0.78	0.074	0.00
Fish & Fruit	0.00	0.074	0.026
Grain & Cattle	0.074	0.074	0.32
Roots & Pigs	0.074	0.074	0.18
Religion			
Outlier	0.065	0.065	0.96
Animisim	0.097	0.065	0.4
Autonomous Communities	0.097	0.065	0.25
Heroes	0.065	0.065	0.83
Mana	0.032	0.097	0.022
Cultural Interaction			
Outlier	0.00	0.00	0.19
Contact	0.00	0.00	0.46
Isolation	0.00	0.00	0.46

Table S12: Correlation of missingness with extreme archetype values. In general the degree of missingness does not have a strong effect on the max/min for a given society and cultural class.

	ρ (Max)	P-value (Max)	ρ (Min)	P-value (Min)
Social Organization	0.08	0.37	-0.01	0.91
Subsistence	0.10	0.24	0.075	0.394
Religion	-0.21	0.013	0.14	0.10
Cultural Interaction	0.037	0.67	-0.037	0.67

Table S13: *Religion* traits sorted by phylogenetic signal over the linguistic phylogeny from Gray et al. (2009). P-values are from LRT with null assumption $\lambda = 0$.

Variable	Definition	Pagel's λ	Log Likelihood	P-value
Pul8	Primordial pair	1.00	-38.6	1E-13
Pul36	Headhunting	0.517	-62.0	4E-05
Pul21	Mana as a spiritual or religious concept	0.497	-60.1	1E-07
Pul84	Religious Authority	0.394	-148.9	0.003
Pul18	Mana related to social influence or technical skill	0.392	-60.4	2E-05
Pul2	Belief in god(s)	0.389	-133.6	0.0005
Pul3	Belief in nature god(s)	0.379	-130.6	0.009
Pul10	The actions of others after one has died can affect the nature of one's afterlife	0.339	-93.6	0.0002
Pul35	Costly sacrifices and offerings	0.330	-29.3	0.0115
Pul4	Belief in deified ancestor(s)	0.309	-148.6	0.0037
Pul37	Political and religious differentiation (SCCS v 757)	0.298	-109.9	0.0118
Pul20	Mana as a personal quality	0.288	-67.4	0.0007
Pul41	Tattooing	0.288	-121.3	0.0388
Pul38	Largest religious community	0.281	-126.5	0.004
Pul85	Largest religious community	0.250	-152.4	0.0240
Pul5	Belief in ancestral spirits	0.214	-115.6	0.107
Pul39	Genital cutting	0.209	-140.4	0.3
Pul11	Own actions affect own afterlife	0.164	-90.6	0.5
Pul14	Social hierarchy tapu	0.151	-63.9	0.108
Pul42	Scarification	0.094	-80.7	0.5
Pul16	Resource management tapu	0.083	-72.7	0.4
Pul12	Belief in culture hero(es)	0.034	-128.6	0.7
Pul7	Belief in supernatural punishment for impiety	0.025	24.0	0.7
Pul6	Belief in nature spirits	7E-05	-121.8	1
Pul9	Myth of humanity creation	7E-05	-119.9	1
Pul15	Kinship tapu	7E-05	-20.5	1
Pul40	Tooth pulling	7E-05	-117.5	1
Pul43	Piercing	7E-05	-97.8	1

References

T. K. Earle. *How Chiefs Come to Power: The Political Economy in Prehistory*. Stanford University Press, Stanford, CA, 1997.

Gary M. Feinman and Joyce Marcus, editors. *Archaic States*. Advanced Seminar Series. School for Advanced Research Press, Santa Fe, NM, 1998.

Norman Yoffee. *Myths of the Archaic State: Evolution of the Earliest Cities, States, and Civilizations*. Cambridge University Press, Cambridge and New York, 2005.

Kathryn R. Kirby et al. D-place: A global database of cultural, linguistic and environmental diversity. *PLoS ONE*, 11(7):e0158391, 2016.

Laura Fortunato. Lineal kinship organization in cross-specific perspective. *Phil Trans Roy Soc B*, 374 (1780):20190005, 2019.

F. Pedregosa, G. Varoquaux, A. Gramfort, V. Michel, B. Thirion, O. Grisel, M. Blondel, P. Prettenhofer, R. Weiss, V. Dubourg, J. Vanderplas, A. Passos, D. Cournapeau, M. Brucher, M. Perrot, and E. Duchesnay. Scikit-learn: Machine learning in Python. *Journal of Machine Learning Research*, 12:2825–2830, 2011.

Anonymous. Details omitted for double anonymised reviewing. *In progress methods paper inclusion*, 2023.

D Justin Yeh, Laurel Fogarty, and Anne Kandler. Cultural linkage: the influence of package transmission on cultural dynamics. *Proceedings of the Royal Society B*, 286(1916):20191951, 2019.

Matthew Spriggs. The Lapita culture and Austronesian prehistory in Oceania. *The Austronesians*, 119, 1995.

W Arthur Whistler. Plants of the canoe people. *Hawai'i: National Tropical Botanical Garden*, 2009.

Lucienne Wilme, Patrick O Waeber, and Joerg U Ganzhorn. Marine turtles used to assist Austronesian sailors reaching new islands. *Comptes rendus biologies*, 339(2):78–82, 2016.

Alfredo González-Ruibal. War between neighbours: the archaeology of internal conflict and civil war, 2019.

Omer Karin and Uri Alon. Tradeoffs and cultural diversity. *bioRxiv*, page 263905, 2018.

Jason R Ali and Lawrence R Heaney. Wallace's line, wallacea, and associated divides and areas: history of a tortuous tangle of ideas and labels. *Biological Reviews*, 96(3):922–942, 2021.

Lixia Ju, Huijun Wang, and Dabang Jiang. Simulation of the last glacial maximum climate over east asia with a regional climate model nested in a general circulation model. *Palaeogeography, Palaeoclimatology, Palaeoecology*, 248(3-4):376–390, 2007.

Penny C Gardner, Satyawan Pudyatmoko, Naris Bhumpakphan, Marnoch Yindee, Datuk Laurentius N Ambu, and Benoit Goossens. Banteng bos javanicus d'alton, 1823. *Ecology, evolution and behaviour of wild cattle: implications for conservation*, pages 216–230, 2014.

Peter Bellwood et al. *Austronesian prehistory in Southeast Asia: homeland, expansion and transformation*. Canberra: ANU E Press, 1995.

Russell D Gray, Alexei J Drummond, and Simon J Greenhill. Language phylogenies reveal expansion pulses and pauses in Pacific settlement. *Science*, 323(5913):479–483, 2009.

Oliver Sheehan, Joseph Watts, Russell D Gray, Joseph Bulbulia, Scott Claessens, Erik J Ringen, and Quentin D Atkinson. Coevolution of religious and political authority in Austronesian societies. *Nature Human Behaviour*, 7(1):38–45, 2023.

Jeannette Mageo. Myth, cultural identity, and ethnopolitics: Samoa and the Tongan "empire". *Journal of Anthropological Research*, 58(4):493–520, 2002.

Niel Gunson. The Tonga-Samoa connection 1777–1845: Some observations on the nature of Tongan imperialism. *The Journal of Pacific History*, 25(2):176–187, 1990.

Paul Geraghty. Linguistic evidence for the Tongan Empire. In Tom Dutton and Darrell T. Tryon, editors, *Language Contact and Change in the Austronesian World*, Trends in Linguistics, Studies and Monographs [TILSM], pages 233–249. Mouton de Gruyter, Berlin and New York, 1994.

Richard Scaglion. Kumara in the Ecuadorian Gulf of Guayaquil. In C. Ballard, P. Brown, R.M. Bourke, and T. Harwood, editors, *The Sweet Potato in Oceania: A Reappraisal*, pages 35–41. University of Sydney Press, Pittsburgh and Sydney, 2005.

Geoffrey K Chambers and Hisham A Edinur. Reconstruction of the Austronesian diaspora in the era of genomics. *Human Biology*, 92(4):247–263, 2021.

Alexander G Ioannidis, Javier Blanco-Portillo, Karla Sandoval, Erika Hagelberg, Carmina Barberena-Jonas, Adrian VS Hill, Juan Esteban Rodríguez-Rodríguez, Keolu Fox, Kathryn Robson, Sonia Haoa-Cardinali, et al. Paths and timings of the peopling of Polynesia inferred from genomic networks. *Nature*, 597(7877):522–526, 2021.

J Watts, O Sheehan, S J Greenhill, S Gomes-Ng, Q D Atkinson, J Bulbulia, et al. Pulotu: Database of Austronesian supernatural beliefs and practices. *PLoS ONE*, 10(9):e0136783, 2015.

Aaron A Behr, Katherine Z Liu, Gracie Liu-Fang, Priyanka Nakka, and Sohini Ramachandran. Pong: fast analysis and visualization of latent clusters in population genetic data. *Bioinformatics*, 32(18): 2817–2823, 2016.

S. T. Morris. Economics of sheep production. *Small Ruminant Research*, 86(1-3):59–62, 2009.

Sarah Rehkamp, Michael J Roberts, and James M MacDonald. The agricultural economic landscape in Hawaii and the potential for future economic viability. Policy Brief of the Economic Research Organization of the University of Hawaii (UHERO), 2021.

Michael E Moseley and Robert A Feldman. Fishing, farming, and the foundations of andean civilisation. *The archaeology of prehistoric coastlines*, pages 125–134, 1988.

Joyce Marcus. The archaeological evidence for social evolution. *Annual Review of Anthropology*, 37: 251–266, 2008.

David Beresford-Jones, Alexander Pullen, George Chauca, Lauren Cadwallader, Maria García, Isabel Salvatierra, Oliver Whaley, Víctor Vásquez, Susana Arce, Kevin Lane, et al. Refining the maritime foundations of Andean civilization: how plant fiber technology drove social complexity during the preceramic period. *Journal of Archaeological Method and Theory*, 25:393–425, 2018.

Patrick V Kirch, Anthony S Hartshorn, Oliver A Chadwick, Peter M Vitousek, David R Sherrod, James Coil, Lisa Holm, and Warren D Sharp. Environment, agriculture, and settlement patterns in a marginal polynesian landscape. *Proceedings of the National Academy of Sciences*, 101(26): 9936–9941, 2004.

Patrick Vinton Kirch. *On the Road of the Winds*. University of California Press, Oakland, 2017.

Ronald L Trosper. Resilience in pre-contact pacific northwest social ecological systems. *Conservation Ecology*, 7(3), 2003.

Michael Edward Moseley. *The Maritime Foundations of Andean Civilization*. Cummings Publishing Company, Menlo Park, CA, 1975.

Michael E Moseley. Maritime foundations and multilinear evolution: Retrospect and prospect. *Andean Past*, 3(1):5, 1992.

Michael E Moseley. The maritime foundations of andean civilization: an evolving hypothesis. *Submitted August*, 10:2004, 2005.

Alan R Templeton. *Population genetics and microevolutionary theory*. John Wiley & Sons, 2021.
